# Myeloid Cell Arg1 Inhibits Control of Arthritogenic Alphavirus Infection by Suppressing Antiviral T Cells

**DOI:** 10.1371/journal.ppat.1005191

**Published:** 2015-10-05

**Authors:** Kristina S. Burrack, Jeslin J. L. Tan, Mary K. McCarthy, Zhisheng Her, Jennifer N. Berger, Lisa F. P. Ng, Thomas E. Morrison

**Affiliations:** 1 Department of Immunology and Microbiology, University of Colorado School of Medicine, Aurora, Colorado, United States of America; 2 Singapore Immunology Network, Agency for Science, Technology, and Research, Singapore; Mount Sinai School of Medicine, UNITED STATES

## Abstract

Arthritogenic alphaviruses, including Ross River virus (RRV) and chikungunya virus (CHIKV), are responsible for explosive epidemics involving millions of cases. These mosquito-transmitted viruses cause inflammation and injury in skeletal muscle and joint tissues that results in debilitating pain. We previously showed that arginase 1 (Arg1) was highly expressed in myeloid cells in the infected and inflamed musculoskeletal tissues of RRV- and CHIKV-infected mice, and specific deletion of Arg1 from myeloid cells resulted in enhanced viral control. Here, we show that Arg1, along with other genes associated with suppressive myeloid cells, is induced in PBMCs isolated from CHIKV-infected patients during the acute phase as well as the chronic phase, and that high Arg1 expression levels were associated with high viral loads and disease severity. Depletion of both CD4 and CD8 T cells from RRV-infected Arg1-deficient mice restored viral loads to levels detected in T cell-depleted wild-type mice. Moreover, Arg1-expressing myeloid cells inhibited virus-specific T cells in the inflamed and infected musculoskeletal tissues, but not lymphoid tissues, following RRV infection in mice, including suppression of interferon-γ and CD69 expression. Collectively, these data enhance our understanding of the immune response following arthritogenic alphavirus infection and suggest that immunosuppressive myeloid cells may contribute to the duration or severity of these debilitating infections.

## Author Summary

Mosquito-transmitted chikungunya virus (CHIKV), Ross River virus (RRV), and related alphaviruses cause epidemics involving millions of persons, such as on-going CHIKV outbreaks in the Caribbean and Central and South America. Infection with these viruses results in severe pain due to inflammation of musculoskeletal tissues that can persist for months and even years. There are no specific therapeutics or licensed vaccines for these viruses. Suppressive myeloid cells have been shown to inhibit anti-pathogen immune responses, including T cell responses, which can promote chronic disease. We showed previously that a gene associated with suppressive myeloid cells, arginase 1 (Arg1), was induced in musculoskeletal tissues and macrophages of mice infected with RRV or CHIKV, and mice that lacked Arg1 expression in myeloid cells had reduced viral loads at late times post-infection. Here, we demonstrate that Arg1 is induced in PBMCs isolated from CHIKV-infected patients, and Arg1 expression is associated with viral loads. Moreover, we found that Arg1-expressing myeloid cells inhibit the activation and function of antiviral T cells in RRV-infected mice. These studies underscore the role of suppressive myeloid cells in modulating the T cell response to arthritogenic alphaviruses and provide a therapeutic target to enhance viral clearance and potentially limit chronic disease.

## Introduction

Arthritogenic alphaviruses, including chikungunya virus (CHIKV) and Ross River virus (RRV), are re-emerging, mosquito-transmitted alphaviruses that cause both endemic and explosive epidemics of debilitating musculoskeletal inflammatory disease [1]. CHIKV has caused outbreaks of unprecedented scale involving millions of persons in the Indian Ocean Islands [2], India [3], Southeast Asia [4,5,6] and Europe [7]. Most recently, CHIKV has emerged in the Western Hemisphere where ongoing epidemics on multiple islands in the Caribbean as well as in Central and South America have resulted thus far in more than one million suspected cases [8,9,10]. RRV, which causes ~4,000–7,000 cases in Australia and Papua New Guinea annually, has similarly caused explosive outbreaks [11]. For example, an RRV epidemic occurred in 1979–1980 with > 60,000 cases, where RRV spread from Australia to multiple islands in the Pacific Region including Fiji, the Cook Islands, and America Samoa [12,13,14]. Currently there are no specific therapies for the treatment of alphavirus-induced rheumatological disease and no licensed vaccines.

CHIKV/RRV-induced disease is characterized by fever, intense pain and inflammation in joints, tendons, and muscles, and an impaired ability to ambulate [11]. This acute stage lasts for 1 to 2 weeks and is typically followed by convalescence. However, some disease signs and symptoms—such as joint swelling, joint stiffness, arthralgia, and tendonitis/tenosynovitis—can last for months to years, with up to 60% of patients reporting persistent rheumatological symptoms three years after initial diagnosis [15,16,17,18,19,20,21]. This chronic phase of the disease has been linked in both humans and animal models to persistent CHIKV/RRV infection in the affected musculoskeletal tissues [22,23,24,25].

Monocytes and macrophages can be activated by a variety of stimuli, resulting in a spectrum of activation phenotypes [26]. Macrophages that promote tissue repair/remodeling during wound healing and have immunoregulatory functions express arginase 1 (Arg1), an enzyme that hydrolyzes L-arginine [27]. High Arg1 expression has been associated with a variety of diseases such as chronic inflammation [28], asthma [29], and infectious diseases [30,31,32,33,34]. The expression of Arg1 by human and murine monocytes/macrophages, neutrophils, and myeloid-derived suppressor cells (MDSCs) has emerged as a major regulator of immune responses [35,36,37]. Indeed, Arg1 activity in myeloid cells impairs effective immunity against intracellular pathogens such as *Mycobacterium tuberculosis* and *Toxoplasma gondii* [38], exacerbates tumor growth by suppressing T cell function [39,40], and limits T cell-driven inflammatory tissue damage [41]. Arg1 activity has also been associated with higher viral loads and lower CD4^+^ T cell counts from HIV-seropositive patients [33,42] and with inhibition of CD8^+^ T cell responses in hepatitis B virus (HBV) and hepatitis C virus (HCV)-infected patients [34,43].

We previously showed that Arg1 was induced in the musculoskeletal inflammatory lesions and tissue-infiltrating macrophages of RRV- and CHIKV-infected mice [44]. We further showed that mice specifically deleted for Arg1 in myeloid cells had reduced viral loads, as well as improved tissue pathology, at late, but not early, times post-RRV infection, indicating that Arg1^+^ macrophages prevent efficient host control of RRV infection in musculoskeletal tissues [44]. We sought to expand our previous findings in the mouse model to human CHIKV infections. Here, we show that Arg1 is induced in peripheral blood mononuclear cells (PBMCs) from CHIKV-infected patients and that higher Arg1 expression levels were associated with higher viral loads and more severe disease. Using the RRV and CHIKV mouse models, we further investigated the mechanism(s) by which Arg1 regulates clearance of arthritogenic alphavirus infection. We found that Arg1-expressing myeloid cells inhibited the antiviral T cell response in RRV-infected mice, resulting in reduced expression of the antiviral cytokine interferon (IFN)-γ and modulation of T cell activation markers.

## Results

### Arg1 is induced in PBMCs from CHIKV-infected patients and Arg1 expression levels are associated with viral load and disease severity

**Fig 1:**
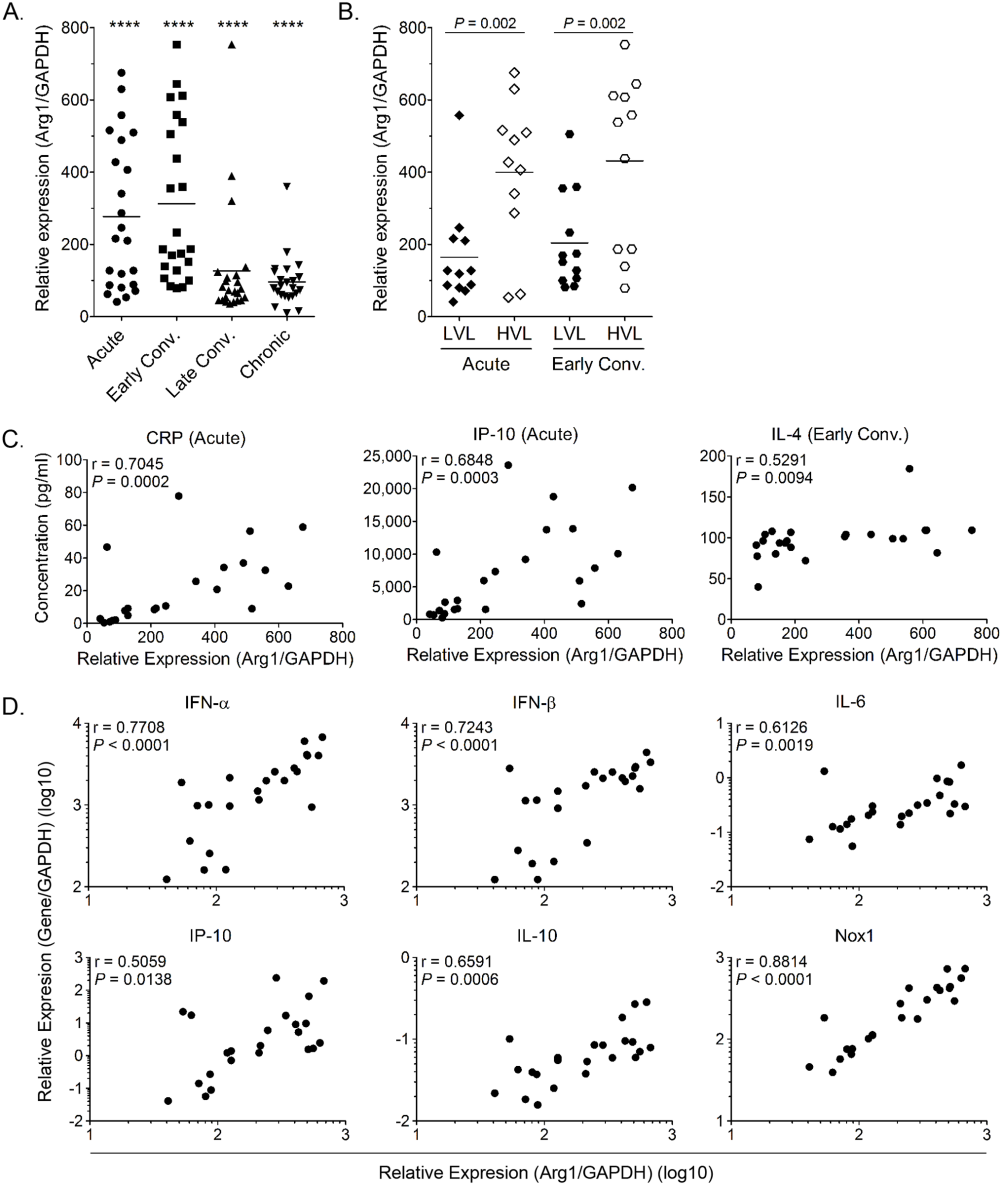
Arg1 is significantly induced in PBMCs collected from CHIKV-infected patients and expression correlates with viral load. (A) PBMCs isolated from CHIKV-infected patients (n = 23) were analyzed for Arg1 gene expression compared to healthy controls (n = 8). At each time point analyzed, Arg1 expression was found to be statistically different from healthy controls (**** *P* < 0.001), as determined by performing a two-tailed Mann Whitney *U* test. Each dot represents a patient, and the horizontal line represents the mean. (B) Based on their viremia during the acute and early convalescent phase of infection, patients were grouped into either a low viral load group (LVL; n = 11) or a high viral load group (HVL; n = 12). Arg1 expression was compared between the two groups via two-tailed Mann Whitney *U* test. * *P* < 0.05. (C and D) Spearman’s correlation analysis between transcriptional profiles of Arg1 expression in PBMCs of CHIKV-infected patients (n = 23) and (C) acute plasmatic C-reactive protein (CRP) and IP-10 levels, and IL-4 plasma levels during the early convalescent phase as well as (D) IFN-α, IFN-β, IL-6, IP-10, IL-10, and Nox1 genes during acute disease. The level of Arg1 gene expression was expressed relative to healthy controls (n = 8) after normalization to GAPDH. Early Conv., early convalescent phase.

We have previously shown that Arg1 is highly induced in inflamed and infected musculoskeletal tissues and infiltrating macrophages of mice infected with RRV or CHIKV [44]. Moreover, mice specifically deleted for Arg1 in myeloid cells had reduced viral loads at late, but not early, times post-RRV infection, suggesting that Arg1 activity in macrophages inhibits efficient host control of RRV infection in musculoskeletal tissues [44]. To evaluate the role of Arg1 during CHIKV infection in patients, we analyzed the gene expression profile of PBMCs collected at four times post-illness onset (PIO) in a cohort of CHIKV-infected patients in Singapore: (1) acute phase (median, 4 days PIO); (2) early convalescent phase (median, 10 days PIO); (3) late convalescent phase (4–6 weeks PIO); and (4) chronic phase (2–3 months PIO) [45]. Compared to healthy controls, we found that *Arg1* expression levels were significantly increased in circulating PBMCs during the acute phase and remained elevated into the chronic phase of the disease (Fig 1A). By segregating the cohort into high and low viral load groups (HVL and LVL, respectively), as previously defined [45], higher Arg1 expression was observed to associate with higher viral loads in these patients (Fig 1B). High viremia was also previously shown to correlate with increased disease severity during the acute phase of infection (ref. [45] and S1A Fig). By segregating on disease severity, we were able to analyze Arg1 gene expression in PBMCs isolated from patients with mild or severe clinical disease throughout the disease time course. We found that although Arg1 expression levels were equally elevated in PBMCs isolated from patients with mild or severe disease during the acute, early convalescent, and chronic phases of disease (S1B, S1C and S1E Fig), PBMCs isolated from patients with severe disease during the late convalescent phase (4–6 weeks PIO) expressed Arg1 at significantly higher levels than PBMCs isolated from patients with mild disease (*P* = 0.002) (S1D Fig). Due to the low number of patients that complained of persistent arthralgia 2–3 months PIO in this study (6 of 23 patients), there was no association between Arg1 expression levels in PBMCs isolated from patients with persistent join pain during the chronic phase of disease (2–3 months PIO) compared to those who had fully recovered.

In addition to comparing Arg1 expression levels to viral loads and clinical disease, we compared Arg1 gene expression in PBMCs to plasma concentrations of C-reactive protein (CRP) and to the expression of other genes in PBMCs collected during the acute phase (median, 4 days PIO) or early convalescent phase (median, 10 days PIO). Positive correlations were observed between Arg1 gene expression and the plasma concentration of CRP, IP-10, and IL-4 (Fig 1C) as well as the gene expression of type I interferons (IFNs), IL-6, IP-10, IL-10, and NADPH oxidase 1 (Nox1) in PBMCs isolated during the acute phase (Fig 1D). This suggests that these factors are induced as part of the early response to CHIKV infection. Overall, these data show that, consistent with our studies in mice, Arg1 expression is induced in PBMCs isolated from humans infected with an arthritogenic alphavirus, and Arg1 expression levels are associated with viral load and disease severity. Additionally, other genes that have been shown to have suppressive functions, such as Nox1, or to be associated with polarizing suppressive myeloid cells, such as IL-6, are also elevated in PBMCs from CHIKV-infected patients. In sum, these data suggest that suppressive myeloid cells may be induced in human CHIKV infection.

### CHIKV infection of human fibroblasts *in vitro* induces Arg1 in human monocytes

**Fig 2:**
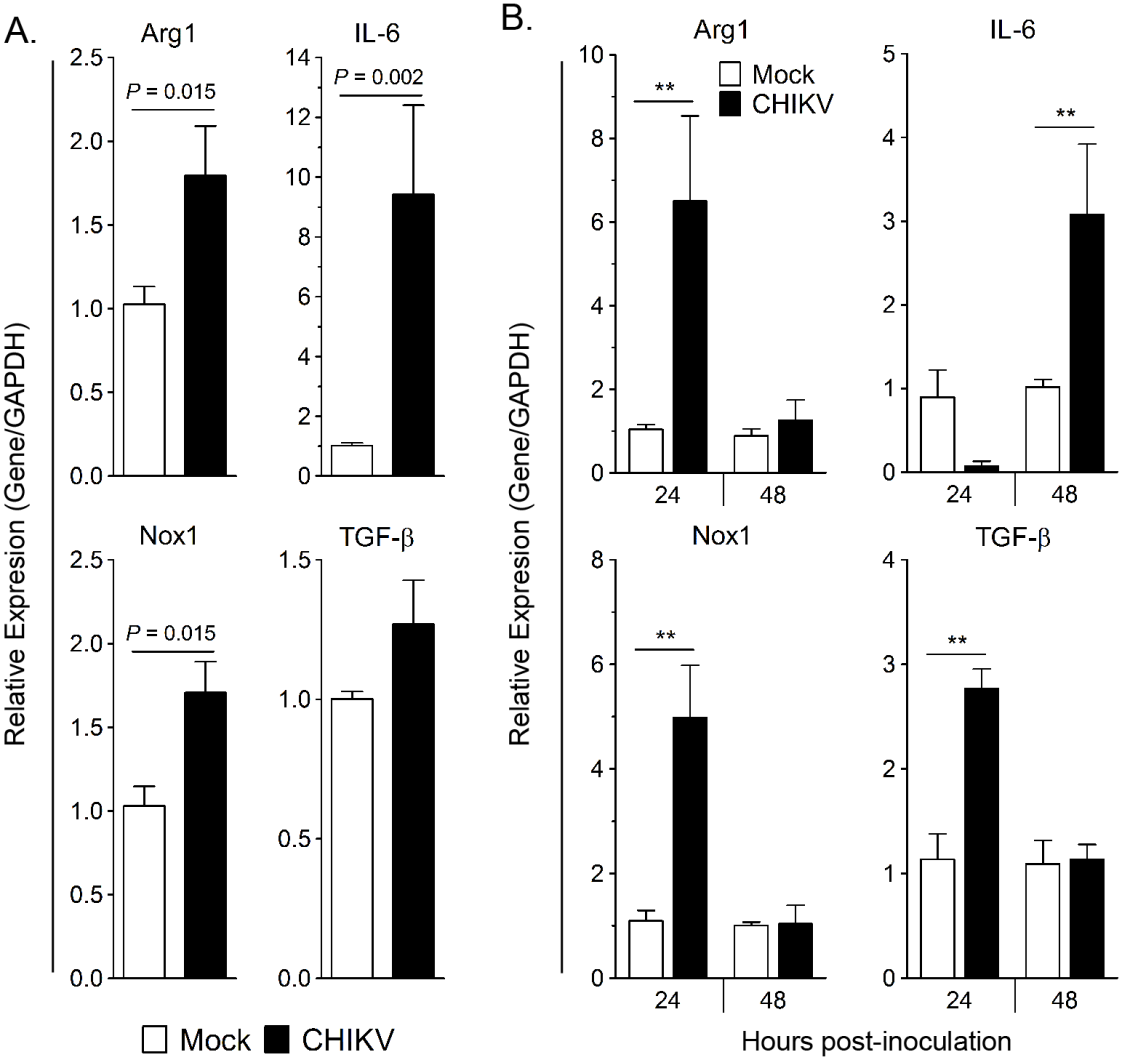
CHIKV-infected HS 633T fibroblast co-culture and supernatant induces *Arg1* expression in human monocytes. (A) CHIKV adsorption (MOI 10) on HS 633T cells was performed for 1.5 h before virus overlay was removed; human monocytes were then co-cultured with the HS 633T cells for 24h. The level of gene expression was expressed as fold change compared to monocytes co-cultured with mock-infected cells after normalization to GAPDH. Experiments were performed in triplicate and data are mean ± SEM of two independent donors. Pair-wise comparison was performed using a two-tailed Mann Whitney *U* test. (B) HS 633T cells were infected with CHIKV (MOI 10) for 24h and 48h. Cell-free supernatant were collected and overlaid onto human monocytes for 24h. The level of gene expression was expressed as fold change compared to monocytes inoculated with cell-free supernatant from mock-infected cell lines after normalization to GAPDH. Experiments were performed in triplicate, and data are represented as mean ± SEM of two independent donors. Pair-wise comparison was performed using two-tailed Mann Whitney *U* test (** *P* < 0.01).

Since Arg1 is inducible in myeloid cells, we sought to test if exposure to CHIKV would induce Arg1 or other genes with immunoregulatory functions in human monocytes *ex vivo*. To do this we utilized the human fibrosarcoma cell line HS 633T, which has been shown to be susceptible to CHIKV infection [46]. To recapitulate the *in vivo* setting whereby monocytes infiltrate infected joint and muscle tissue, human monocytes isolated from PBMCs via negative selection were co-cultured with mock or CHIKV-infected HS 633T cells for 24 hpi, at which time all of the cells were harvested for gene expression analysis. The transcriptional profile of these groups was compared to mock or CHIKV-infected fibroblasts cultured in the absence of monocytes. Expression of the Arg1, IL-6, and Nox1 genes was significantly upregulated in the CHIKV-infected co-cultures (Fig 2A). These data suggest that CHIKV-infection of human fibroblasts *in vitro* induces a transcriptional profile in co-cultured human monocytes that has features similar to myeloid suppressor cells.

Next, we sought to determine if direct contact was required to induce Arg1 expression in monocytes. Monocytes were isolated from PBMCs and stimulated for 24 hours with supernatant from mock- or CHIKV-inoculated HS 633T cells collected at 24 hpi. Virus present in the cell culture supernatant was not inactivated or removed prior to incubation with monocytes. Arg1, TGF-β, and Nox1 transcripts were induced in the monocytes cultured in the CHIKV-infected cell supernatant compared to those cultured in culture supernatants collected from the mock-inoculated cells (Fig 2B). Following 24 h culture with fibroblast supernatant harvested at 48 hpi, IL-6 expression was significantly upregulated in the monocytes (Fig 2B). These data indicate that direct contact with infected fibroblasts is not required for Arg1 induction in monocytes; instead, soluble factor(s), which could include virus and/or other soluble factors such as cytokines, in the supernatant mediate activation of this transcriptional profile.

To investigate if the presence of live virus in cell culture supernatants from alphavirus-infected cells is required for the induction of Arg1 in myeloid cells, we performed experiments utilizing J774 murine macrophages and C2C12 myoblasts that had been differentiated into myofibers to mimic muscle cells, which are a target cell of RRV and CHIKV infection *in vivo* (S2 Fig). In these studies, supernatant transfer experiments were performed to determine whether RRV-infected muscle cells produce factors that induce the expression of Arg1 in macrophages. Stimulation of J774 macrophages with IL-4 (a positive control) or culture supernatants from RRV-infected differentiated C2C12 muscle cells induced Arg1 expression (representative blot in S2A Fig, quantified in S2B Fig). In contrast, stimulation of macrophages with cell culture supernatants from mock-infected differentiated C2C12 muscle cells did not induce Arg1 expression above levels detected in control cells. To determine if the virus present in these cell culture supernatants was required for Arg1 induction in macrophages, we stimulated J774 macrophages with cell supernatant that was left untreated, treated with ultraviolet light (UV) or heat to inactivate the virus, or ultracentrifuged to eliminate virus. Additionally, macrophages were treated with virus that was purified via density gradient centrifugation. The ultra-centrifugation, UV, and heat inactivation treatments of the C2C12 cell culture supernatants effectively reduced the amount of live virus (S2D Fig). Regardless of the presence of live or inactivated virus, the infected cell supernatant induced Arg1 expression in the macrophages, whereas purified virus did not (representative blot in S2C Fig, quantified in S2E Fig). These data suggest that a factor(s) present in culture supernatants other than the virus itself mediates induction of Arg1 expression.

### Arg1 is induced at the sites of inflammation and in the blood, but not in lymphoid tissues

**Fig 3:**
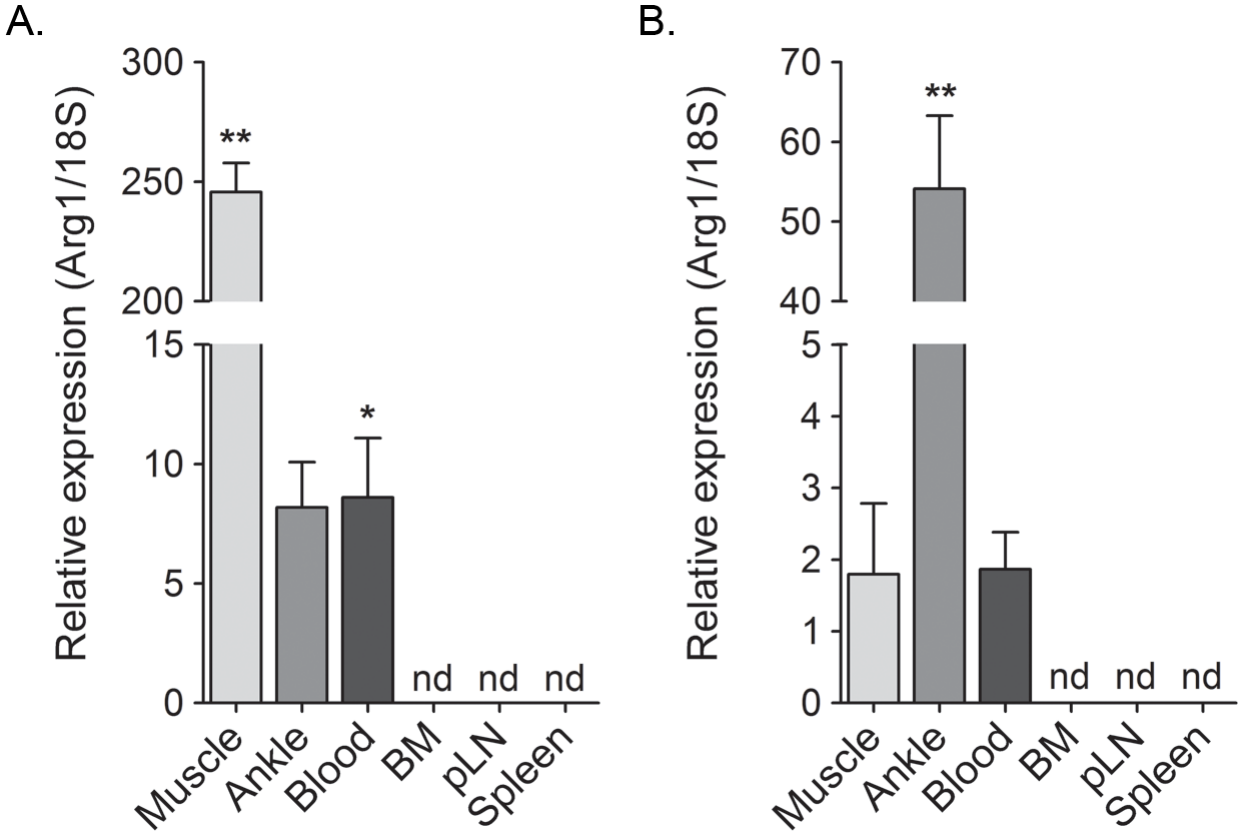
Arg1 is induced in inflamed musculoskeletal tissues and in blood leukocytes but not lymphoid tissues of RRV- and CHIKV-infected mice. WT mice were mock-inoculated (n = 5) or inoculated with 10^3^ PFU of (A) RRV (n = 6) or (B) CHIKV (n = 6). At 10 dpi the gastrocnemius muscle (“Muscle”), ankle/foot joint (“Joint”), circulating blood leukocytes (“Blood”), bone marrow (“BM”) from the femur, the draining popliteal LN (“pLN”), and the spleen were harvested for RT-qPCR analysis of *Arg1* expression. Data are combined from two independent experiments, normalized to 18S rRNA levels, and are expressed as the relative expression (*n*-fold increase) over expression in each tissue from mock-inoculated mice. Each data point represents the arithmetic mean ± SEM. ** *P* < 0.01, * *P* < 0.05 as determined by significance test of greater than one.

Previously, we showed that Arg1 is expressed in infiltrating macrophages present in musculoskeletal tissues of CHIKV- or RRV-infected mice [44]. Since exposure of primary human monocytes to supernatants collected from CHIKV-infected cells induced Arg1 expression (Fig 2), we sought to further define the tissue compartments in which Arg1 was induced in CHIKV- and RRV-infected mice. The gastrocnemius muscle, circulating blood leukocytes, bone marrow, spleen, and draining popliteal lymph node were harvested from mock-, RRV-, or CHIKV-inoculated mice at 7 dpi for RT-qPCR analysis of Arg1. As demonstrated previously [44], Arg1 transcript was highly induced in inflamed muscle tissue of RRV-infected mice (245-fold increase, *P* = 0.002) as well as ankle joint tissue (8.2-fold increase, *P* = 0.06) (Fig 3A). Additionally, Arg1 was induced in circulating blood leukocytes (8.6-fold increase, *P* = 0.02). In contrast, Arg1 expression was not induced in lymphoid tissues such as bone marrow cells, cells from the draining popliteal LN, or spleen cells (Fig 3A). Similar results were observed in tissues collected from CHIKV-infected mice, although the highest level of Arg1 induction was observed in ankle-joint associated tissue (54-fold increase, *P* = 0.002) as opposed to the gastrocnemius muscle in RRV-infected mice (Fig 3B). These data indicate that, similar to our analysis of Arg1 in PBMCs from CHIKV-infected humans, Arg1 expression was elevated in circulating cells from CHIKV- and RRV-infected mice, albeit to a lesser extent.

### Arg1-expressing myeloid cells inhibit antiviral T cells

**Fig 4:**
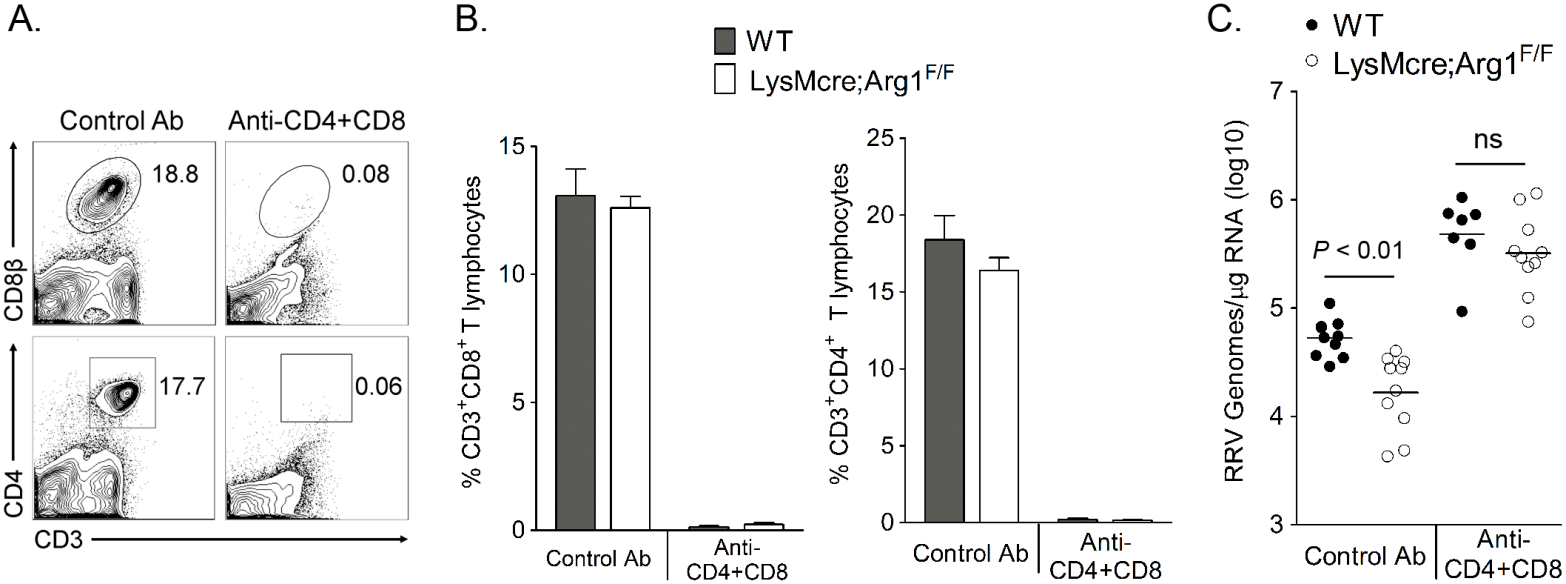
T cell depletion from LysMcre;Arg1^F/F^ mice restores RRV loads to those in depleted WT mice at 14 dpi in muscle tissue. Three-to-four week-old WT or LysMcre;Arg1^F/F^ mice were inoculated with 10^3^ PFU of RRV and treated with anti-CD4 and anti-CD8α antibodies (WT, n = 7; LysMcre;Arg1^F/F^, n = 10) or a control antibody (n = 10 per genotype) on days 7 and 12 pi. At 14 dpi, spleen cells were stained for CD3, CD4, and CD8β to determine the efficiency of T cell depletion. (A) Representative flow plots demonstrating efficient depletion of CD8^+^ (top panel) and CD4^+^ (bottom panel) T cells compared to the control antibody-treated mice. (B) Frequency of CD3^+^CD8^+^ (left panel) and CD3^+^CD4^+^ (right panel) T cells in the spleens of WT and LysMcre;Arg1^F/F^ mice. Data are represented as the arithmetic mean ± SEM. (C) At 14 dpi, the right quadriceps muscle was dissected, total RNA was isolated, and RRV genomes were quantified by absolute RT-qPCR. Horizontal bars indicate the mean. Data are combined from 2–3 independent experiments. *P*-value was determined by one-way ANOVA followed by Tukey’s multiple comparison test.

Arg1-expressing myeloid cells have immune-suppressive activity [41,47,48]. Additionally, we have previously shown that Arg1-expressing CD11b^+^F4/80^+^ macrophages present in the inflamed musculoskeletal tissues of RRV-infected mice suppressed T cell proliferation *ex vivo* in a dose-dependent manner by a mechanism that was partially Arg1-dependent [44]. Furthermore, mice specifically deleted for Arg1 in myeloid cells (LysMcre;Arg1^F/F^ mice) have significantly lower viral loads in musculoskeletal tissues, suggesting that Arg1 activity in macrophages prevents efficient host control of RRV infection in musculoskeletal tissues [44]. Based on these data, we hypothesized that tissue-infiltrating myeloid cells inhibited RRV clearance by suppression of the antiviral T cell response. To investigate this hypothesis, WT and LysMcre;Arg1^F/F^ mice were treated with anti-CD4 and anti-CD8α antibodies, or an isotype control antibody, on days 7 and 12 pi to deplete CD4^+^ and CD8^+^ T cells. Experiments were terminated at 14 dpi, spleens were analyzed for efficiency of T cell depletion, and muscle tissue was harvested for absolute quantification of RRV genome levels. Administration of anti-CD4 and anti-CD8α antibodies depleted ≥ 98% of both CD4^+^ and CD8^+^ T cell subsets as demonstrated by staining spleens for CD3, CD4, and CD8β (Fig 4A and 4B). As with untreated mice [44], control antibody-treated LysMcre;Arg1^F/F^ mice had significantly lower viral RNA levels compared to control antibody-treated WT mice (3.2-fold decrease, *P* < 0.05) (Fig 4C). The difference between control antibody-treated LysMcre;Arg1^F/F^ mice and WT mice is not as substantial as we previously published in untreated LysMcre;Arg1^F/F^ mice and WT mice [44]; this may be due to unanticipated effects of the isotype control antibody treatments. Interestingly, following depletion of both CD4^+^ and CD8^+^ T cells, LysMcre;Arg1^F/F^ mice had similar viral loads to T cell-depleted WT mice (*P* > 0.05) (Fig 4C). Taken together, these data suggest that Arg1-expressing myeloid cells have suppressive activity and inhibit antiviral T cells.

### Mice deficient in myeloid cell Arg1 have an increased number of virus-specific CD8^+^ T cells in inflamed muscle tissue following RRV infection

One mechanism by which myeloid cell Arg1 has been shown to suppress T cells is by inhibiting T cell proliferation [47,49]. To determine if myeloid cell Arg1 inhibits T cell stimulation and proliferation in lymphoid tissues, T cell populations in the spleen and draining popliteal lymph node (LN) were analyzed by flow cytometry at 7, 10, and 14 days post-RRV inoculation of WT and LysMcre;Arg1^F/F^ mice. After gating on lymphocytes, CD8^+^ T cells were identified by staining positive for CD3 and CD8; CD4^+^ T cells were identified by gating on CD3^+^CD8^-^ cells followed by gating on CD3^+^CD4^+^ cells (S3A Fig). We found that WT and LysMcre;Arg1^F/F^ mice had similar frequencies and total numbers of CD4^+^ and CD8^+^ T cells in both the spleen and draining popliteal LN at all of the time points analyzed (S3B–S3E Fig).

**Fig 5:**
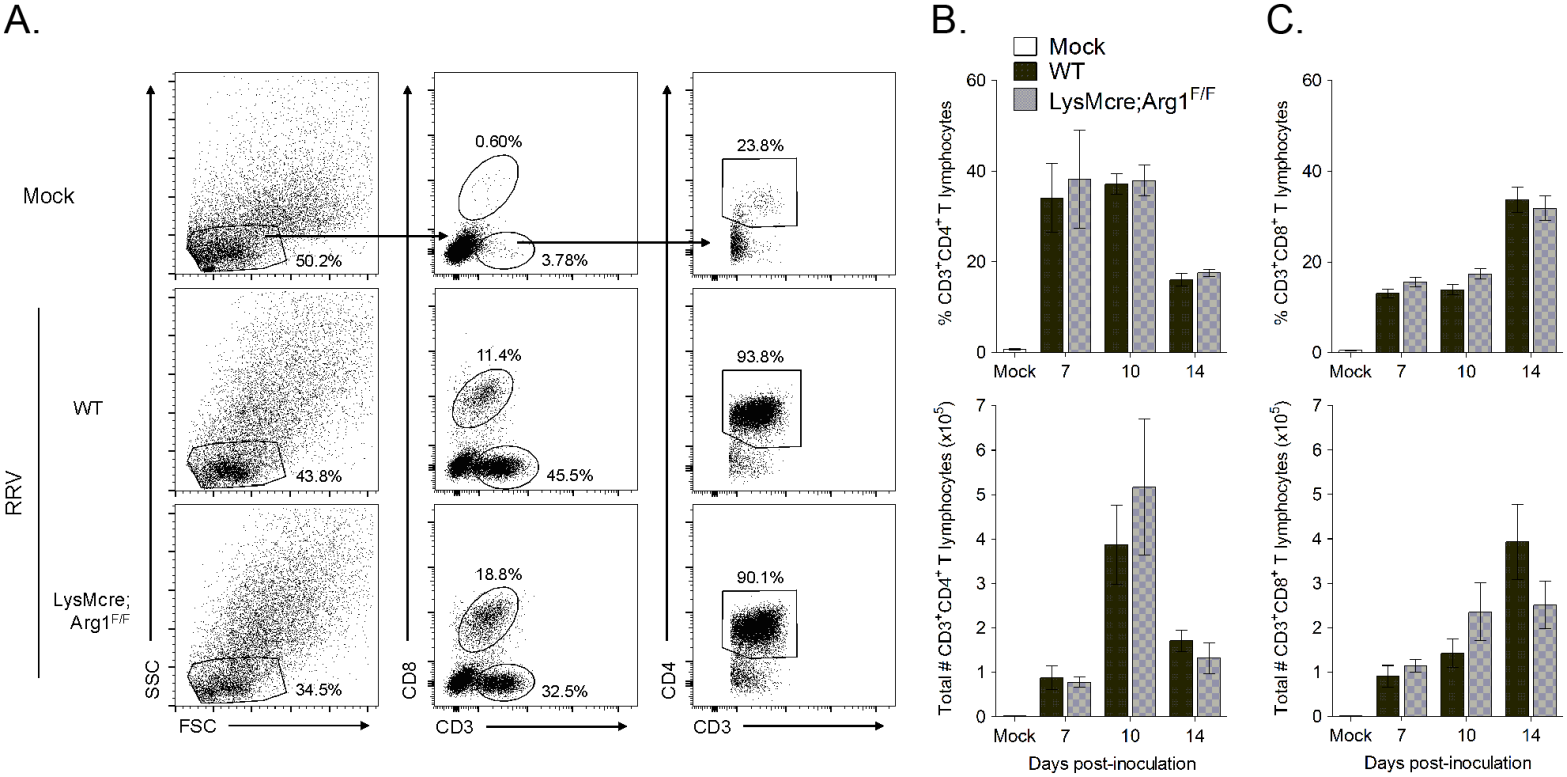
Similar frequencies and total number of CD4^+^ and CD8^+^ T cells in inflamed muscle tissues of WT and LysMcre;Arg1^F/F^ mice at 7, 10, and 14 dpi. Three-to-four week-old WT and LysMcre;Arg1^F/F^ mice were mock-inoculated (n = 3) or inoculated with 10^3^ PFU of RRV (n = 7–8 per time point per genotype). At 7, 10, and 14 dpi, quadriceps muscles were dissected, enzymatically digested, and the infiltrating leukocytes were isolated for FACS analysis. (A) Representative flow plots indicating the gating strategy to identify lymphocytes, CD3^+^CD8^+^ T cells versus CD3^+^CD8^-^ T cells, and CD3^+^CD4^+^ T cells in the muscle tissue of mock (top panel), RRV-inoculated WT (middle panel), and RRV-inoculated LysMcre;Arg1^F/F^ (bottom panel) mice at 10 dpi. Frequency (top panel) and total number (bottom panel) of (B) CD3^+^CD4^+^ T cells and (C) CD3^+^CD8^+^ T cells in muscle tissue of RRV-inoculated WT and LysMcre;Arg1^F/F^ mice compared with mock-inoculated mice. Data are represented as the arithmetic mean ± SEM and combined from two independent experiments. Each time point was individually evaluated for statistical difference by a two-tailed, unpaired *t*-test, and all were found to be not significant (*P* > 0.05).

Since Arg1 is highly induced in inflamed musculoskeletal tissues but not lymphoid tissues of RRV-infected mice (ref. [44] and Fig 3), we hypothesized that the inhibitory effects of Arg1-expressing myeloid cells could be localized to sites of pathology in the musculoskeletal tissues. Thus, we next assessed the presence of CD4^+^ and CD8^+^ T cells in inflamed muscle tissue of WT versus LysMcre;Arg1^F/F^ mice at 7, 10, and 14 dpi. CD4^+^ and CD8^+^ T cells were identified as described above (Fig 5A). We again found similar frequency and number of total CD4^+^ and CD8^+^ T cells in the quadriceps muscle of both WT and LysMcre;Arg1^F/F^ mice at all time points analyzed (Fig 5B and 5C). In total, these data suggest that although myeloid cell Arg1 inhibits the antiviral T cell response, this is not through the suppression of total T cell numbers at immune inductive sites or the sites of infection and inflammation.

**Fig 6:**
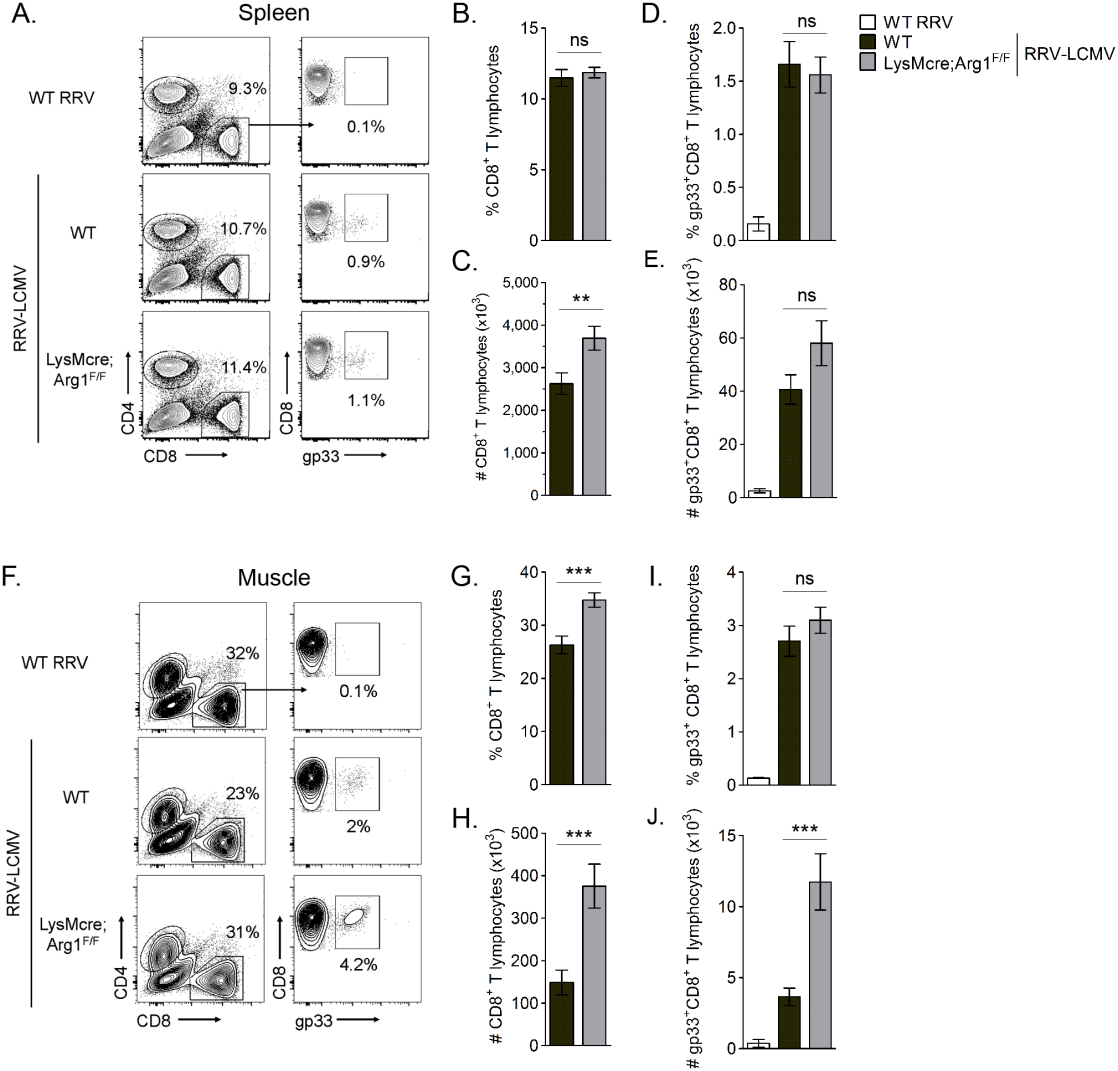
LysMcre;Arg1^F/F^ mice have significantly more virus-specific CD8^+^ T cells in inflamed muscle tissue than WT mice at 10 dpi. Three-to-four week-old WT and LysMcre;Arg1^F/F^ mice were inoculated with 10^3^ PFU of WT RRV (n = 3) or recombinant RRV-LCMV (n = 18 for WT mice; n = 19 for LysMcre;Arg1^F/F^ mice). At 10 dpi, leukocytes from (A-E) spleens and (F-J) quadriceps muscles (following enzymatic digestion) were isolated for FACS analysis of virus-specific CD8^+^ T cells. Muscle-infiltrating leukocytes from a mouse inoculated with WT RRV was used as a control for gp33 tetramer staining. (A, F) Representative flow plots demonstrating the gating strategy to identify gp33^+^CD8^+^CD4^-^ T cells in the spleen (A) and muscle (F) tissue at 10 dpi. Frequency (B, G) and total number (C, H) of CD8^+^CD4^-^ T cells in the spleen (B, C) and muscle (G, H) tissue of WT RRV-inoculated mice, RRV-LCMV-inoculated WT mice, and RRV-LCMV-inoculated LysMcre;Arg1^F/F^ mice at 10 dpi. Frequency (D, I) and total number (E, J) of gp33^+^CD8^+^CD4^-^ T cells in the spleen (D, E) and muscle (I, J) tissue of WT RRV-inoculated mice, RRV-LCMV-inoculated WT mice, and RRV-LCMV-inoculated LysMcre;Arg1^F/F^ mice at 10 dpi. Data are represented as the arithmetic mean ± SEM and combined from five independent experiments. *** *P* < 0.001 as determined by two-tailed, unpaired *t*-tests with or without Welch’s correction (WT versus LysMcre;Arg1^F/F^).

Arg1-mediated suppression has been shown to inhibit antigen-specific T cell responses [50]. For example, in *Leishmania*-infected mice, parasite-specific T cell proliferation was suppressed at the site of pathology, where high arginase activity was detected, but not in the draining LN, where arginase activity was low or undetectable [48]. Thus, we next quantified virus-specific T cell responses in both lymphoid tissues and inflamed muscle tissue of RRV-infected mice. For these experiments we utilized a recombinant Ross River virus (“RRV-LCMV”) that was recently developed in our laboratory [51] and encodes a CD8 (gp33) TCR immunodominant epitope of LCMV, enabling the identification and quantification of virus-specific CD8^+^ T cells in tissues via tetramer staining (see Materials and Methods for description of virus generation). WT and LysMcre;Arg1^F/F^ mice were inoculated with RRV-LCMV and gp33^+^CD8^+^ T cells were analyzed by flow cytometry at 10 dpi, a time point associated with extensive skeletal muscle inflammation and damage as well as high Arg1 expression [44]. To confirm the specificity of the gp33 tetramer staining, leukocytes from the spleens of mock- and WT RRV-infected mice were also stained for gp33-specific T cells. Similar to the analysis of bulk CD8^+^ T cells in WT RRV-infected mice (S3 Fig), we found that the total number of CD8^+^ T cells in the spleen of RRV-LCMV-infected LysMcre;Arg1^F/F^ mice was mildly elevated in comparison to WT mice (Fig 6C). However, the total number of virus antigen-specific gp33^+^CD8^+^ T cells in the spleen of RRV-LCMV-infected LysMcre;Arg1^F/F^ mice was similar to WT mice (Fig 6E). Since Arg1 is highly induced in the inflamed muscle tissue of RRV-infected mice (Fig 3 and ref. [44]), we next analyzed the bulk and virus-specific CD8 T cell response in muscle tissue of WT and LysMcre;Arg1^F/F^ mice. The gp33^+^ gate was set such that ≤ 0.1% of CD8^+^ T cells from the muscle tissue of a WT RRV-inoculated mouse stained positive with the gp33 tetramer (Fig 6F). Interestingly, RRV-LCMV-infected LysMcre;Arg1^F/F^ mice had significantly increased frequency and number (2.5-fold) of CD8^+^ T cells in the muscle tissue compared to RRV-LCMV-infected WT mice (Fig 6G and 6H). This suggests that loss of myeloid cell Arg1 may have a more profound effect on CD8 T cells in the presence of a RRV expressing the CD8 T cell immunodominant epitope from LCMV, gp33. Indeed, there was a slight but non-significant difference in the frequency of gp33-specific CD8^+^ T cells (Fig 6I); moreover, there was a significantly greater number (3.2-fold) of gp33^+^CD8^+^ T cells detected in the inflamed quadriceps muscle tissue of RRV-LCMV-infected LysMcre;Arg1^F/F^ mice than WT mice (Fig 6J). These data are consistent with the data presented in Fig 3, which shows elevated expression of Arg1 in skeletal muscle tissue but not lymphoid tissue. In addition, these data suggest that loss of myeloid cell Arg1 increased virus-specific T cell trafficking, proliferation, and/or avoidance of deletion at the site of inflammation.

### Muscle-infiltrating T cells from Arg1-deficient mice are more activated following RRV infection

**Fig 7:**
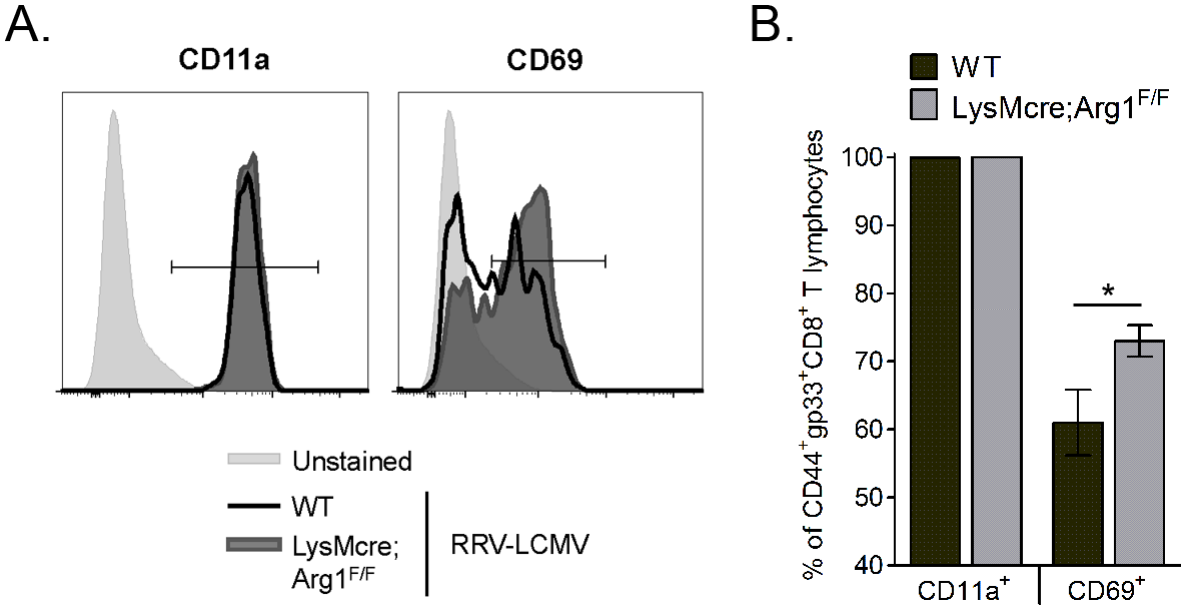
Loss of myeloid cell Arg1 results in activation of virus-specific CD8^+^ T cells in inflamed muscle tissue. Three-to-four week-old WT (n = 8) and LysMcre;Arg1^F/F^ (n = 10) mice were inoculated with 10^3^ PFU of RRV-LCMV. At 10 dpi, leukocytes from quadriceps muscles (following enzymatic digestion) were isolated for flow cytometric analysis of CD11a and CD69 expression on virus-specific CD8^+^ T cells. Muscle-infiltrating leukocytes that were left unstained were utilized as a control for CD11a and CD69 staining. (A) Representative histograms demonstrating CD11a (left) and CD69 (right) expression on CD44^+^gp33^+^CD8^+^CD4^-^ T cells at 10 dpi. (B) Frequency of CD11a^+^ or CD69^+^ cells (of CD44^+^gp33^+^CD8^+^CD4^-^ T cells) in the muscle tissue of RRV-LCMV-inoculated WT mice and LysMcre;Arg1^F/F^ mice at 10 dpi. Data are represented as the arithmetic mean ± SEM and combined from two independent experiments. * *P* < 0.05 as determined by two-way ANOVA followed by a Bonferroni multiple comparison test.

In addition to inhibiting T cell proliferation and trafficking, Arg1-expressing myeloid cells can also inhibit T cell activation [36,37]. We next sought to investigate the activation of virus-specific CD8^+^ T cell responses in WT and LysMcre;Arg1^F/F^ mice. The three activation markers that we evaluated are known to be upregulated on antigen-experienced T cells: CD44, CD11a, and CD69 [52]. Of the CD44^+^gp33^+^CD8^+^ T cells in WT and LysMcre;Arg1^F/F^ mice, essentially all were also CD11a^+^ (representative histogram shown in Fig 7A; quantified in Fig 7B). However, significantly more CD44^+^gp33^+^CD8^+^ T cells in LysMcre;Arg1^F/F^ mice stained for CD69 than those cells in WT mice (representative histogram shown in Fig 7A; quantified in Fig 7B), suggesting specific modulation of this activation marker on virus-specific T cells in the absence of arginase activity. These data suggest that the inflamed musculoskeletal tissue environment alters the T cell activation phenotype in an Arg1-dependent manner.

**Fig 8:**
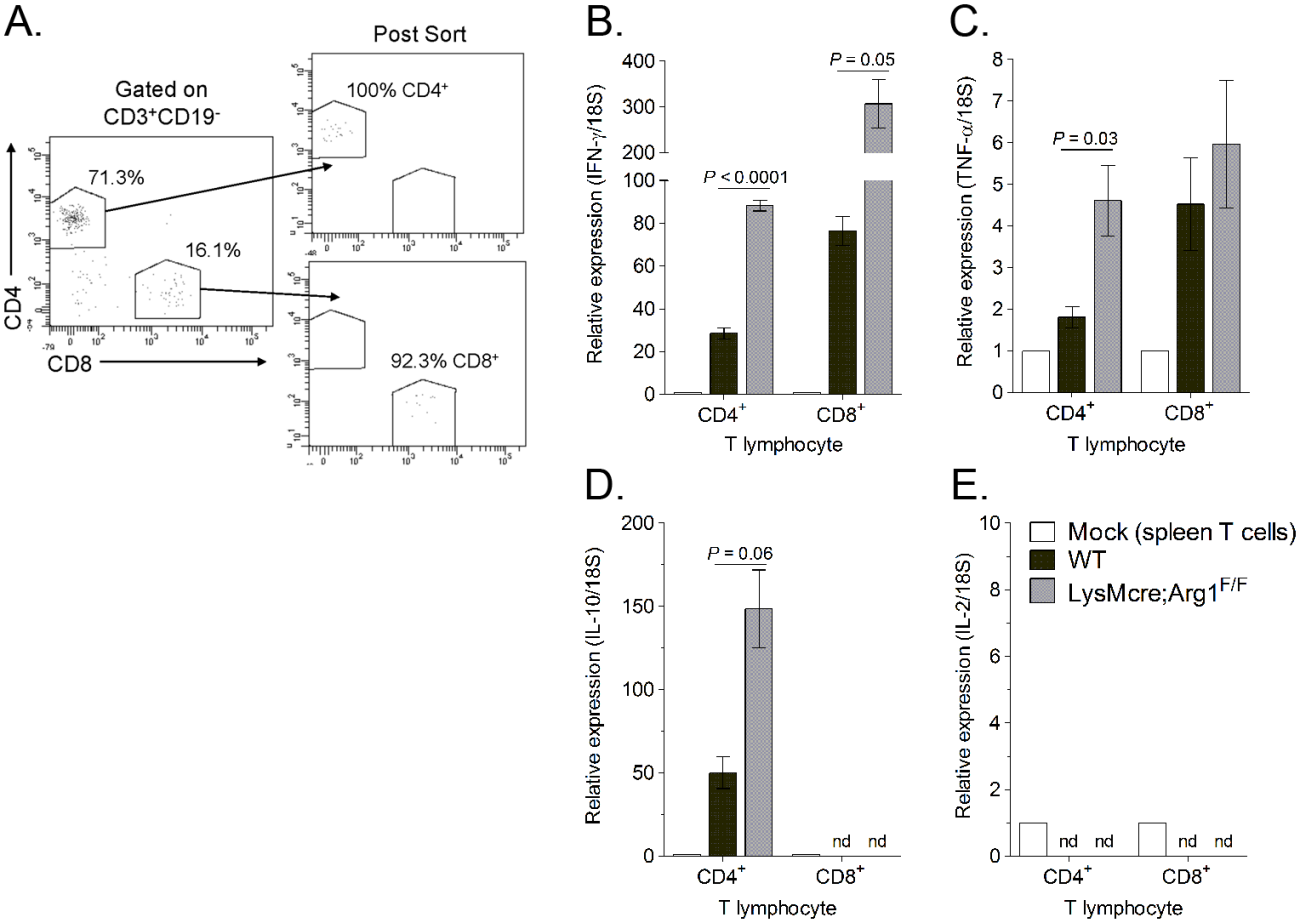
Muscle-infiltrating CD4^+^ and CD8^+^ T cells isolated from LysMcre;Arg1^F/F^ mice express enhanced IFN-γ transcript. (A) Representative flow plots demonstrating the gating strategy to FACS-sort CD19^-^CD3^+^CD4^+^CD8^-^ and CD19^-^CD3^+^CD8^+^CD4^-^ T cells isolated from quadriceps muscles of RRV-infected WT or LysMcre;Arg1^F/F^ mice at 10 dpi and the post-sort purities. RT-qPCR analysis of (B) IFN-γ, (C) TNF-α, (D) IL-10, and (E) IL-2 expression in FACS-sorted T cells from the spleen of a mock-inoculated mouse (n = 1) or from the quadriceps muscle of RRV-infected WT (n = 3) or LysMcre;Arg1^F/F^ mice (n = 3) at 10 dpi. Data are normalized to 18S rRNA levels and are expressed as the relative expression (*n*-fold increase) over expression in spleen T cells from a mock-inoculated mouse. Data are represented as the arithmetic mean ± SEM. Data are representative of two independent experiments. *P*-values determined by two-tailed, unpaired *t*-tests with or without Welch’s correction. nd, not detected.

To further explore the effects of Arg1 on T cell function, we investigated the cytokine expression levels in T cells sorted from muscle tissue of RRV-infected WT and LysMcre;Arg1^F/F^ mice. At 10 dpi, quadriceps muscle tissue was dissected and enzymatically digested, and infiltrating leukocytes were stained for CD3, CD19, CD4, and CD8. CD4^+^ and CD8^+^ T cells were FACS-sorted by gating on CD3^+^CD19^-^ cells and then gating individually on CD4^+^ T cells and CD8^+^ T cells (Fig 8A). Gene expression analysis of the sorted T cell subsets was compared to respective T cells sorted from the spleen of a mock-infected mouse. Both CD4^+^ and CD8^+^ T cell subsets isolated from LysMcre;Arg1^F/F^ mice expressed increased levels of IFN-γ compared to T cells sorted from WT mice (Fig 8B). T cells sorted from the spleens of RRV-infected WT or LysMcre;Arg1^F/F^ mice showed no difference in IFN-γ expression (S4 Fig). CD4^+^ T cells sorted from LysMcre;Arg1^F/F^ mice also expressed elevated levels of TNF-α (Fig 8C) and IL-10 (Fig 8D) transcripts. In contrast, CD8^+^ T cells sorted from WT or LysMcre;Arg1^F/F^ mice expressed similar levels of TNF-α, and IL-10 expression was not detected in this T cell subset. Additionally, IL-2 expression was not detected in either T cell subset (Fig 8E). To further confirm cytokine expression, T cells from RRV-infected WT mice isolated at 10 dpi were restimulated *ex vivo* with anti-CD3 and anti-CD28 antibodies followed by intracellular cytokine staining. A subset of CD4^+^ T cells produced both IFN-γ and IL-10, whereas CD8^+^ T cells produced IFN-γ but very little IL-10 (S5 Fig). These data suggest that Arg1 activity in macrophages inhibits cytokine expression, including IFN-γ, by T cells in musculoskeletal tissues, which may be one mechanism by which Arg1 influences viral loads.

Suppressive myeloid cells have been shown to mediate T cell suppression through the induction or expansion of regulatory T (Treg) cells [53], which can indirectly inhibit CD8^+^ T cells. Interestingly, we found that at 10 days post-RRV infection a subset of muscle-infiltrating CD4^+^ T cells produce IL-10 (Fig 8D and S5 Fig), a cytokine that has anti-inflammatory functions and has been shown to regulate Arg1 expression levels in myeloid cells [53]. To determine if CD4^+^ T cells were contributing to Arg1 induction in musculoskeletal tissues following RRV infection, we treated mice with an anti-CD4 Ab or a control Ab on day 4 pi and harvested muscle tissues on day 7 pi for analysis of CD4 T cell numbers, macrophage numbers, RRV loads, and Arg1 expression. Administration of anti-CD4 Ab depleted ≥ 95% of CD4^+^ T cells in the spleen and muscle tissue as demonstrated by staining for CD3 and CD4 (S6A–S6E Fig). Importantly, the depletion of CD4^+^ T cells did not reduce the total number of macrophages present in skeletal muscle tissue (S6A, S6F and S6G Fig). Mice that were depleted of CD4^+^ T cells had reduced Arg1 expression in muscle tissue at 7 dpi compared to control Ab-treated mice (146-fold increase versus 271-fold increase), however these differences were not statistically significant (S6H Fig). Consistent with viral loads in LysMcre;Arg1^F/F^ mice at 7 dpi, mice depleted of CD4^+^ T cells had similar viral loads as control Ab-treated mice (S6I Fig). These data suggest that Arg1 expression levels in tissues are predominantly regulated by a CD4^+^ T cell-independent mechanism(s).

### IFN-γ expression by T cells is required to control RRV infection in muscle tissue of infected *Rag1* ^-/-^ mice

**Fig 9:**
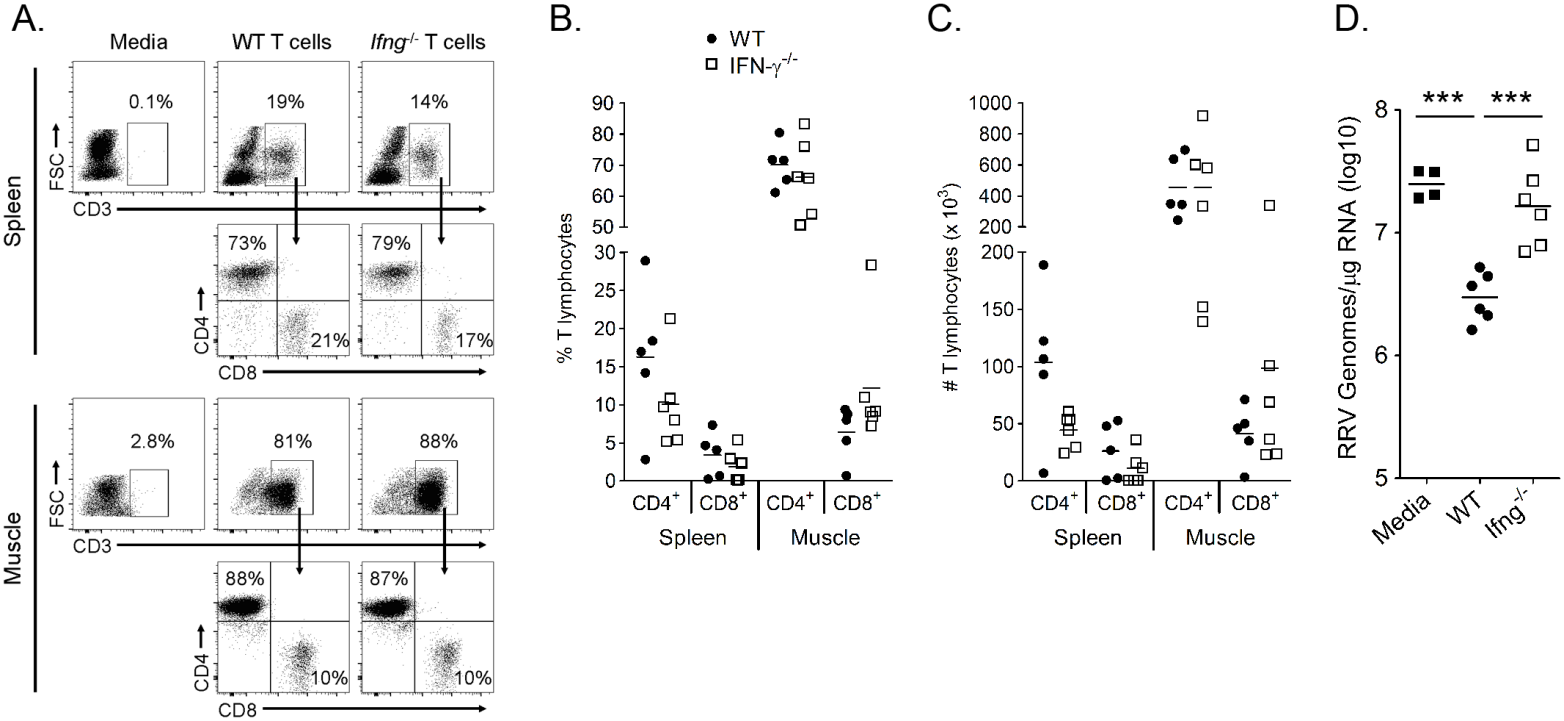
IFN-γ expression by T cells is required to control RRV infection in *Rag1* ^-/-^ mice. T cells were isolated from the spleens of 6–8 week-old WT and *Ifng* ^-/-^ mice via negative selection and 2.5 x 10^6^ T cells were adoptively transferred into three-to-four week-old *Rag1* ^-/-^ mice i.p. one day prior to inoculation with 10^3^ PFU of RRV. (A-C) At 14 dpi, spleen and left quadriceps muscle tissues were harvested for flow cytometric analysis of the presence of T cells. (A) Representative flow plots showing the frequency of CD3^+^ and CD4^+^ or CD8^+^ T cells in the spleen and muscle tissues of *Rag1* ^-/-^ mice that received either WT T cells or *Ifng* ^-/-^ T cells. Quantification of the (B) frequency and (C) total number of CD4^+^CD3^+^ or CD8^+^CD3^+^ T cells in the mice that received T cells. Horizontal bars indicate the mean. (D) At 14 dpi, the right quadriceps muscle was dissected, total RNA was isolated, and RRV genomes were quantified by absolute RT-qPCR. Horizontal bars indicate the mean. Data are combined from 2 independent experiments. *P*-value was determined by a two-way, unpaired *t-*test.

Other groups have shown that IFN-γ production from T cells was critical for clearance of SINV RNA from neurons [54], demonstrating a role for IFN-γ-mediated clearance of alphavirus RNA. To further investigate the role for T cell-derived IFN-γ in control of RRV infection, we adoptively transferred T cells from WT and *Ifng* ^-/-^ mice into *Rag1* ^-/-^ mice, which lack B and T cells, one day prior to RRV inoculation and analyzed RRV RNA levels at 14 dpi in muscle tissues compared to *Rag1* ^-/-^ mice that received media alone. T cell reconstitution was confirmed by flow cytometric analysis of spleen and muscle tissue at 14 dpi for the presence of cells staining positively for CD3 and CD4 or CD3 and CD8 and negatively for B220 and GR-1 (Fig 9A). Although T cell engraftment varied between individual mice, the frequencies (Fig 9B) and total numbers (Fig 9C) of CD4 and CD8 T cells in the spleen and muscle tissue of mice receiving WT or *Ifng* ^-/-^ T cells were comparable. RRV RNA levels were lower in muscle tissue of mice that received WT T cells compared to mice that received media alone (9-fold decrease, *P* < 0.001) (Fig 9D). Moreover, RRV RNA levels in *Rag1* ^-/-^ mice that received *Ifng* ^-/-^ T cells were similar to mice that received no T cells, which was significantly greater than RRV levels in *Rag1* ^-/-^ mice that received WT T cells (7.1-fold increase, *P* < 0.001) (Fig 9D). These data indicate that IFN-γ production is critical for the antiviral effects of T cells following RRV infection. In sum, our data suggest that Arg1-mediated inhibition of T cell activation and IFN-γ production results in enhanced viral loads, perhaps contributing to viral persistence and chronic disease in humans.

## Discussion

We previously showed that RRV and CHIKV infection in mice resulted in elevated expression of Arg1 in inflamed musculoskeletal tissues and tissue-infiltrating macrophages [44]. Here, we demonstrate that Arg1 is also highly induced in PBMCs isolated from CHIKV-infected patients during the acute phase and remained elevated in PBMCs isolated from patients 2–3 months post-illness onset. These data suggest that CHIKV infection may result in the expansion of immunoregulatory myeloid cells that express high levels of Arg1. Moreover, higher Arg1 transcript levels were associated with higher viral loads and with more severe disease, suggesting a relationship between viral levels, disease severity, and cells that express Arg1. Immune-suppressive myeloid cells are heterogeneous populations of myeloid cells that are functionally defined by their potent ability to suppress T cell functions via a variety of mechanisms, including Arg1 activity [53]. We previously demonstrated that specific ablation of *Arg1* in myeloid cells enhanced the clearance of RRV from musculoskeletal tissues and diminished muscle tissue pathology at late times post-RRV infection [44]. Here, we show that the enhanced control of RRV infection in LysMcre;Arg1^F/F^ mice is likely due to more effective antiviral T cell responses. Several lines of evidence support this conclusion. First, in our previous study we found that CD11b^+^F4/80^+^ macrophages sorted from muscle tissue of RRV-infected mice suppressed T cell proliferation *ex vivo* in a mixed leukocyte reaction via a mechanism that was partially Arg1-dependent [44]. Here, depletion of CD4^+^ and CD8^+^ T cells from WT and LysMcre;Arg1^F/F^ mice increased viral loads compared to the respective control antibody-treated mice at 14 dpi in inflamed muscle tissue, consistent with our previous studies that demonstrated a role for T cells in controlling RRV infection in muscle tissue [51]. However, viral loads in the muscle tissue of T cell-depleted WT and LysMcre;Arg1^F/F^ mice at 14 dpi were not significantly different, suggesting that Arg1 activity in macrophages inhibited the antiviral activity of T cells at the sites of infection.

Arginase-expressing myeloid cells can inhibit T cell functions via a variety of mechanisms, including reducing the bioavailablity of L-arginine, increasing the production of reactive nitrogen species such as peroxynitrite, and expanding regulatory T cell populations [53]. For instance, depletion of extracellular L-arginine levels from the local microenvironment via the activity of myeloid cell Arg1 has been shown to suppress T cells, including inhibiting T cell proliferation and suppressing other T cell functions such as IFN-γ production [47,49]. We found that WT and LysMcre;Arg1^F/F^ mice had similar frequencies and numbers of CD4^+^ and CD8^+^ T cells in the spleen and draining popliteal LN at 7, 10, and 14 dpi, suggesting that myeloid cell Arg1 did not affect T cell numbers in lymphoid tissues. These data are consistent with our findings showing that Arg1 expression is highly induced in inflamed musculoskeletal tissues but not lymphoid tissues of RRV- and CHIKV-infected mice. This expression pattern is similar to mice infected with the *Leishmania major* parasite where high arginase activity was detected at the site of pathology but not in the draining lymph node [48]. Moreover, Arg1-expressing myeloid cell-mediated suppression has been shown to inhibit antigen-specific T cell responses [50], as was shown for *L*. *major*-specific T cells at the site of pathology [48]. Thus, we hypothesized that virus-specific T cells, rather than bulk T cells, would be inhibited specifically at the site of pathology in musculoskeletal tissue. To this end we found that CD4^+^ and CD8^+^ T cell frequencies and numbers were similar in muscle tissue of WT and LysMcre;Arg1^F/F^ mice at 7, 10, and 14 dpi. However, utilizing a recombinant RRV encoding the CD8 immunodominant epitope of LCMV, we found that LysMcre;Arg1^F/F^ mice, as compared to WT mice, had significantly more virus-specific CD8^+^ T cells in the muscle tissue at 10 dpi, a time point preceding the significant difference in viral loads detected at 14 dpi. Further studies will be required to determine if this is a result of deletion of virus-specific T cells due to depletion of amino acids, the inability to detect gp33-specific T cells at the sites of pathology in WT mice, and/or a result of enhanced virus-specific T cell proliferation in muscle tissue of LysMcre;Arg1^F/F^ mice. Preliminary studies show that a similar proportion of bulk and gp33-specific CD8^+^ T cells in spleen and muscle tissue stain for the proliferation marker Ki67 at 7 dpi (S7 Fig), suggesting that arginase activity may not inhibit T cell proliferation in RRV-infected WT mice.

Alternatively or additionally, an effect of myeloid cell Arg1 on CD4^+^ T cells, such as through the induction or expansion of Treg cells, another mechanism by which MDSCs mediate T cell suppression [53], could indirectly inhibit virus-specific CD8^+^ T cells. If this mechanism of suppression was occurring in the context of RRV or CHIKV infection, loss of CD4^+^ T cells would result in less severe acute disease. Consistent with that hypothesis, *Cd4* ^-/-^ mice are protected from CHIKV-induced arthritis/swelling [55], suggesting that CD4^+^ T cells may have a pathogenic role in acute CHIKV disease. In this study, we found that during RRV infection a subset of muscle-infiltrating CD4^+^ T cells produce IL-10, a cytokine that has anti-inflammatory functions and has been shown to induce Arg1 expression in myeloid cells via signaling through STAT3 [53]. However, depletion of CD4^+^ T cells resulted in minimal effects on Arg1 expression levels in musculoskeletal tissues at 7 days post-RRV infection, suggesting that Arg1 expression levels are primarily regulated by CD4^+^ T cell-independent mechanisms, and studies are ongoing in our laboratory to define the role of specific cytokines, such as IL-10 and IL-6, and other factors in the regulation of Arg1 expression in musculoskeletal tissues of RRV- and CHIKV-infected mice. In addition, further studies are required to determine if other cell(s) besides T cells are also inhibited by Arg1-expressing myeloid cells in the context of arthritogenic alphavirus infection.

In addition to the increased number of gp33-specific CD8^+^ T cells in muscle tissue of RRV-LCMV-infected LysMcre;Arg1^F/F^ mice, we also found that a greater frequency of these muscle-infiltrating virus-specific T cells stained for the activation marker CD69 compared to virus-specific T cells from WT mice. Importantly, no difference in the expression of another activation marker—CD11a—was seen on virus-specific T cells WT and LysMcre;Arg1^F/F^ mice. These data suggest a modulation of specific activation markers on virus-specific CD8^+^ T cells in the presence or absence of arginase activity. CD69 is an early activation marker found on T cells that recently received stimulation through the TCR and has been shown to be persistently expressed at inflammatory foci [56]. We found that a larger proportion of virus-specific CD8^+^ T cells in muscle tissue of LysMcre;Arg1^F/F^ mice were CD69^+^ at 10 dpi, suggesting that a greater number of T cells are restimulated in the muscle tissue of Arg1-deficient but not Arg1-sufficient mice, augmenting their activation and antiviral functions (e.g., IFN-γ production). Additionally, *Cd69* ^−/−^ T cells are not efficiently retained in lymphoid tissues and also fail to establish or sustain tissue residency [57,58]. Thus, arginase activity may inhibit virus-specific T cell retention in musculoskeletal tissues, resulting in reduced viral control. Additional studies are required to delineate the cause(s) of this differential T cell activation in WT versus LysMcre;Arg1^F/F^ mice.

Studies with Sindbis virus (SINV), an alphavirus that causes encephalomyelitis in mice, have shown that mice unable to make antibodies can clear infectious virus from the brain stem and spinal cord but not the brain [54]. This was shown to be due at least in part through the action of IFN-γ produced by both CD4^+^ and CD8^+^ T cells, resulting in site-specific non-cytolytic clearance of virus from the CNS [54]. Consistent with a role for IFN-γ in controlling alphavirus infection, CHIKV-infected IFN-γ^-/-^ mice had increased serum viral RNA levels compared to WT mice [55]. Here, we demonstrated that muscle-infiltrating CD4^+^ and CD8^+^ T cells express IFN-γ transcripts, and IFN-γ expression is higher in T cells isolated from muscle tissue of RRV-infected LysMcre;Arg1^F/F^ mice compared to T cells isolated from muscle tissue of WT mice, suggesting that myeloid cell Arg1 activity inhibits cytokine expression by T cells in inflamed musculoskeletal tissues. Increased IFN-γ mRNA expression combined with an increased frequency of CD69^+^ T cells in musculoskeletal tissues of Arg1-deficient mice suggests that a lack of arginase activity may lead to more efficient T cell restimulation within the inflamed and infected musculoskeletal tissues. This results in more effective antiviral T cells and thus better viral clearance. The mechanism by which IFN-γ acts may include direct antiviral effects as well as regulatory functions important for other immune effector mechanisms, such as increasing expression of MHC class I and class II. Further studies demonstrated that adoptive transfer of naïve WT T cells but not T cells lacking IFN-γ could control RRV infection in muscle tissue of infected *Rag1* ^-/-^ mice, supporting a direct role for IFN-γ. Since uncontrolled cytokine production can be highly toxic, IFN-γ expression by T cells is tightly regulated at the transcriptional level [59]. Indeed, studies with effector CD8^+^ T cells during virus infection have shown that cytokine production terminates immediately following loss of antigen contact but is quickly initiated again after antigen contact is restored [59]. The increased IFN-γ expression by muscle-infiltrating T cells from RRV-infected LysMcre;Arg1^F/F^ mice is another indication that the Arg1-driven immunosuppressive environment inhibits T cell responses.

These studies provide important evidence for the role of Arg1-expressing myeloid cells in the control of arthritogenic alphavirus infection in humans and mice. Sustained expression of Arg1 throughout the course of disease suggests that activation of immunosuppressive myeloid cells may contribute to the duration of disease and/or the development of chronic disease. Thus, therapeutics that target the induction or activity of Arg1 could limit the severity or duration of these debilitating virus-induced diseases.

## Materials and Methods

### Ethics statement

All mouse studies were performed in strict accordance with the recommendations in the Guide for the Care and Use of Laboratory Animals of the National Institutes of Health. All mouse studies were performed at the University of Colorado Anschutz Medical Campus (Animal Welfare Assurance #A 3269–01) using protocols approved by the University of Colorado Institutional Animal Care and Use Committee. All studies were performed in a manner designed to minimize pain and suffering in infected animals. CHIKV human PBMC samples were collected from 23 patients that were admitted to the Communicable Disease Centre at Tan Tock Seng Hospital during the 2008 Singapore CHIKF outbreak. All patients were diagnosed with CHIKF and blood was collected with written informed consent obtained from all participants. The study was approved by the National Healthcare Group’s domain-specific ethics review board (DSRB Reference No. B/08/026).

### Study population

A total of 23 PCR-confirmed CHIKV-positive individuals from the 2008 Singapore CHIKV outbreak were included in this study [45]. Peripheral blood mononuclear cell (PBMC) samples were isolated from patients admitted with acute CHIKV disease to the Communicable Disease Centre at Tan Tock Send Hospital during the outbreak from August 1 to September 23, 2008 in Singapore. For a control group, PBMC samples were isolated during the same time period from 8 healthy volunteers (controls) residing in Singapore [60]. PBMCs from patients and controls were isolated using standard Ficoll-Paque density gradient centrifugation method and stored in -80°C until use. Samples were taken at 4 different time points: (a) acute phase (median 4 days post illness onset), (b) early convalescent phase (median 10 days post illness onset), (c) late convalescent phase (4–6 weeks post illness onset), and (d) chronic phase (2–3 months post illness onset). On the basis of their viral loads, quantified upon admission to hospital, the patients were classified into either high viral load (HVL) group (n = 11) or low viral load (LVL) group (n = 12) [45,60]. Based on the clinical parameters defined in earlier studies [45,61], illness was defined as “severe” if a patient had either a maximum temperature greater than 38.5°C, or a maximum pulse rate greater than 100 beats/min, or a nadir platelet count less than 100 x 10^9^/liter. Patients who did not fulfill these criteria were classified as “mild” [45,61]. Ten (91%) of 11 patients with HVL (median viral load, 9.97 x 10^5^ pfu/mL; range, 1.42 x 10^5^–5.62 x 10^8^ pfu/mL) presented with “severe” clinical illness, compared with 1 (19%) of 12 patients with LVL (median viral load, 2.02 x 10^4^ pfu/mL; range, 1 x 10^2^–5.36 x 10^4^ pfu/mL) (see S1A Fig).

### Viruses

The T48 stain of RRV was isolated from *Aedes vigilax* mosquitoes in Queensland, Australia [62]. Prior to cDNA cloning, the virus was passaged 10 times in suckling mice, followed by two passages on Vero cells [63,64]. The SL15649 strain of CHIKV was isolated from a serum sample collected from a febrile patient in Sri Lanka in 2006. This virus was passaged two times in Vero cells prior to cDNA cloning [23].

Gradient-purified RRV was generated as previously described [65]. Briefly, virus particles were banded on a 60% to 20% discontinuous sucrose gradient by centrifugation at 24,000 rpm for 2.5 h at 4°C in a Beckman SW-28 rotor. Banded virus was collected and centrifuged through 20% sucrose at 24,000 rpm for 6 h 4°C in a Beckman SW-28 rotor. Virus pellets were then resuspended, aliquoted, and stored at −80°C.

The recombinant “RRV-LCMV” was generated by inserting a tandem sequence, similar in design to a sequence inserted in the influenza virus genome that encodes the LCMV CD8 T cell receptor epitope gp_33–41_ (KAVYNFATC) and CD4 T cell receptor epitope gp_61–80_ (GLKGPDIYKGVYQFKSVEFD) [66] in-frame with the RRV structural polyprotein as previously described [51]. Stocks of infectious RRV, RRV-LCMV, or CHIKV (SL15649) were generated from cDNAs and titered by direct plaque assay on BHK-21 cells as previously described [44].

The CHIKV isolate used in the experiments involving HS 633T cells and monocytes was originally isolated from a French patient returning from Reunion Island during the 2006 CHIKV outbreak (IMT strain) [67]. Virus stocks were prepared via numerous passages in Vero-E6 cultures, titered, washed, and precleared by centrifugation before storing at –80°C. These virus stocks were titered by plaque assay on Vero-E6 cells.

### Cells and reagents

The HS 633T human fibrosarcoma cell line is a kind gift of Philippe Gasque and his team at the University of La Réunion. HS 633T cells were grown in DMEM supplemented with 10% FBS. HS 633T cells were inoculated with CHIKV at a MOI of 1 in serum-free medium for 1.5 h. Inoculum was removed and fresh DMEM containing serum was added. Supernatant was harvested 24 h later. Fresh human PBMCs were isolated from whole blood by gradient centrifugation using Ficoll-Paque. Untouched monocytes were isolated using an indirect magnetic labeling system (Monocyte Isolation Kit II, Miltenyi Biotec). Following selection, monocytes were either plated in serum-free IMDM for 1 h to adhere prior to stimulation with HS 633T cell supernatant for 24 hours post-inoculation (hpi) or co-cultured with mock or CHIKV-inoculated HS 633T cells for 24 hpi. Following the 24 h incubation, supernatant was removed and all of the cells were resuspended in TRIzol (Life Technologies) and stored at –80°C prior to RNA isolation.

C2C12 cells (ATCC CRL-1772) were grown in DMEM (Sigma) containing 10% FBS. To differentiate into myotubes, C2C12 myoblasts were plated in 12 well plates in DMEM containing 2% horse serum which was replaced every-other day. Myotubes were inoculated 6 days after plating and cultured for 48 hours in DMEM containing 2% FBS. C2C12 supernatant was collected into 1.5 mL Eppendorf tubes and centrifiuged at 13,000 rpm for 10 min at 4°C to remove any cellular debris. J774A.1 murine macrophages (ATCC TIB-67) were grown in DMEM containing 10% FBS. For co-culture and supernatant transfer experiments, J774A.1 cells were plated in 48 well plates and cultured in DMEM containing 2% FBS. J774A.1 cells treated with recombinant mouse IL-4 (5 ng/ml; R&D Systems) were used as a positive control for Arg1 induction. J774A.1 cells were inoculated with gradient-purified RRV at a multiplicity of infection (MOI) of 10, which is approximately how much virus was present in the RRV-infected C2C12 supernatant. Prior to addition to J774A.1 cells, identical samples of cell supernatant or purified virus were UV-treated for 15 min (short wave) or heat-treated at 56°C for 1 h to inactivate virus, or were centrifuged at 30,000 rpm in a swinging bucket (SW50.1 Beckman rotor) for 4 h at 4°C to remove live virus. J774A.1 cells were cultured in these conditions for 24 hours. The cells were then washed with PBS and resuspended in RIPA buffer [H_2_O containing 50 mM Tris (pH 8.0), 150 mM NaCl, 1% NP-40, 0.5% sodium deoxycholate, 0.1% sodium dodecyl sulfate, and 1x protease inhibitor complex (Sigma)] for protein analysis.

### Western blots

Protein lysates were separated by Tris-HCl–buffered 10% SDS-PAGE, followed by transfer to polyvinylidene difluoride membranes. Membranes were blocked in 5% milk in PBS containing 0.1% Tween and incubated in the appropriate Abs against the indicated proteins. GAPDH expression was used as a loading control. Anti-mouse Arg1 Ab (V-20) was obtained from Santa Cruz; anti-mouse GAPDH Ab (clone 71.1) was obtained from Sigma-Aldrich. Membranes were imaged on a ChemiDoc XRS Plus imager (Bio-Rad), and bands for Arg1 and GAPDH were quantified using Bio-Rad Image Lab software.

### Mice

C57BL/6 wild-type (stock # 000664), *Rag1* ^-/-^ (stock # 002216), LysMcre (stock # 004781), Arg1^F/F^ (stock # 008879), and *Ifng* ^-/-^ (stock # 002287) mice were obtained from The Jackson Laboratory and bred in house. LysMcre;Arg1^F/F^ mice were generated as previously described [44]. Animal husbandry and experiments were performed in accordance with all University of Colorado School of Medicine Institutional Animal Care and Use Committee guidelines. All mouse studies were performed in an animal biosafety level 3 laboratory. Three-to-four week old mice were used. Mice were inoculated in the left rear footpad with 10^3^ PFU of RRV in diluent (PBS/1% bovine calf serum) in a 10-μl volume. Mock-infected animals received diluent alone. Mice were monitored for disease signs and weighed at 24-h intervals. Disease scores were determined by assessing grip strength, hind limb weakness, and altered gait, as previously described [68].

On the termination day of each experiment, mice were sacrificed by exsanguination, blood was collected, and mice were perfused by intracardial injection of 1x PBS. PBS-perfused tissues were removed by dissection and homogenized in TRIzol Reagent (Life Technologies) with a MagNA Lyser (Roche). Alternatively, quadriceps muscles were dissected, minced, and incubated for 1.5 h with vigorous shaking at 37°C in digestion buffer (RPMI 1640, 10% FBS, 15 mM HEPES, 2.5 mg/ml Collagenase Type 1 [Worthington Biochemical], 1.7 mg/ml DNase I [Roche], 1x gentamicin [Life Technologies], 1% penicillin/streptomycin). Following digestion, cells were passed through a 100-μm cell strainer (BD Falcon) and banded on Lympholyte-M (Cedarlane Laboratories) to isolate infiltrating leukocytes. Additionally, spleens and draining popliteal lymph nodes were dissected from mice and passed through a 100-μm cell strainer. Following red blood cell lysis (spleens only), cells were washed in wash buffer (1x PBS, 15 mM HEPES, 1x gentamicin, 1% penicillin/streptomycin), and total viable cells were determined by trypan blue exclusion. Sera samples were titered by direct plaque assay on BHK-21 cells.

### Flow cytometry and T cell depletion

Leukocytes isolated from enzymatically digested tissues were incubated with anti-mouse FcγRII/III (2.4G2; BD Pharmingen) for 20 min on ice to block nonspecific Ab binding and then stained in FACS staining buffer (1x PBS, 2% FBS) with the following Abs: anti-CD3-fluorescein isothiocyanate (FITC, clone 145-2C11), anti-CD3-allophycocyanin (APC, 145-2C11), anti-CD4-PerCP-Cy5.5 or Pacific Blue (RM4-5), anti-CD8α-phycoerythrin (PE, 53–6.7), anti-CD44-Pacific Blue (IM7), anti-CD69-FITC (H1.2F3), anti-B220-PE-Cy7 (RA3-6B2), and anti-CD19-FITC (6D5) (all from BioLegend); anti-CD8β-PE (H35-17.2), and anti-Gr-1-PE-Cy7 (RB6-8C5) (all from eBioscience). gp33-APC H-2D^b^ KAVYNFATM tetramer was kindly provided by the National Institutes of Health Tetramer Core Facility. Cells were fixed overnight in 1% paraformaldehyde and analyzed on an LSR II using FACSDiva software (Becton Dickinson). Further analysis was done using FlowJo Software (Tree Star). Doublets were excluded using side- and forward-scatter height and width parameters.

For intracellular cytokine analysis, wells in a 96-well plate was coated overnight with anti-CD3 Ab (10 μg/mL). Muscle-infiltrating leukocytes from RRV-infected mice were isolated at 10 dpi. 100 μl RPMI containing 7% FBS, Brefeldin A, and anti-CD28 Ab (2 μg/mL) was added to the 96-well plate followed by addition of muscle-infiltrating leukocytes in 100 μl RPMI containing 7% FBS. Cells incubated in the absence of anti-CD3 and anti-CD28 Abs were used as a control. After 5 h incubation, cells were harvested, and incubated with anti-mouse FcγRII/III (2.4G2) for 20 min on ice followed by surface marker staining in FACS buffer for an additional 20 min on ice. After one wash step, cells were fixed and permeabilized in a 1% paraformaldehyde and saponin solution for 15 min at room temperature. Cells were washed with PBS containing saponin and then stained for intracellular cytokines for 45 min on ice in PBS with saponin. Finally, cells were washed 1X with PBS containing saponin, 1X with FACS buffer, and then stored overnight in 1% paraformaldehyde.

The following antibodies from Bio X Cell were used for depletion studies: rat IgG2b control antibody (anti-KLH, clone LTF-2), CD4-depleting antibody (rat IgG2b; clone YTS 191), and CD8-depleting antibody (rat IgG2b; clone YTS 169.4). Mice were treated on days 7 and 12 post-inoculation via the intraperitoneal route (i.p.) with 200 μg of each CD4- and CD8-depleting antibody, or 400 μg of the control antibody, diluted in PBS to a final volume of 150 μl. Depletion efficiency was determined by flow cytometric analysis of spleen tissue at 14 dpi as described in the text for the presence of cells staining positively for CD3 and CD4 or CD8β. In separate experiments, mice were treated i.p. with 200 μg of CD4-depleting antibody or a control antibody diluted in PBS to a final volume of 150 μl on day 4 pi and harvested on day 7 pi. Depletion efficiency was determined by flow cytometric analysis of spleen and quadriceps muscle tissue as described in the text.

### Cell sorting and adoptive transfer

For cell sorting, mice were sacrificed at 10 dpi and the quadriceps muscles were processed as described above. Cells were stained in FACS staining buffer with anti-CD19-FITC, anti-CD3-APC, anti-CD4-Pacific Blue, and anti-CD8α-PE antibodies (BioLegend). Cells were sorted under BSL2 conditions on a FACSAria cytometer using FACSDiva software (Becton Dickinson). CD19^-^CD3^+^ cells were gated on first, then CD4^+^CD8^-^ or CD4^-^CD8^+^ cells were sorted separately. Cells were resuspended in TRIzol (Life Technologies) and stored at –80°C prior to RNA isolation.

For T cell adoptive transfer experiments, T cells were isolated from the spleens of naïve wild-type C57BL/6 mice or *Ifng* ^-/-^ mice via negative selection using a pan-T cell isolation kit (Miltenyi Biotec). Following isolation, cells were counted and 2.5 x 10^6^ T cells were resuspended in RPMI containing 2% FBS in a total volume of 200 μl for i.p. injection into *Rag1* ^-/-^ mice one day prior to RRV infection. Control *Rag1* ^-/-^ mice received 200 μl of media alone. Of the transferred T cells, ≥ 95% of the cells were CD3^+^ and ~60% of the CD3^+^ T cells were CD4^+^ and ~35% were CD8^+^. Additionally, ≤ 1% of CD19^+^ B cells remained in the injected cell preparations. T cell transfer was confirmed by flow cytometric analysis of spleen and muscle tissue at 14 dpi as described above for the presence of cells staining negatively for B220 and Gr-1 and positively for CD3 and CD4 or CD8α.

### Quantitative RT-PCR

For analysis of gene expression in mouse tissue samples or cells, RNA was isolated using a PureLink RNA Mini Kit (Life Technologies), and 1 μg of total RNA was reverse-transcribed using Superscript III (Life Technologies), random oligo(dT) primers, and RNaseOUT. Real-time qPCR experiments were performed using Taqman gene expression assays and a LightCycler 480 (Roche). 18S rRNA was used as an endogenous control to normalize for input amounts of cDNA. The relative fold induction of amplified mRNA were determined by using the Ct method [69].

For analysis of gene expression in human cells, total RNA was extracted using RNeasy Mini Kit (QIAGEN) according to the manufacturer’s instructions. Quantification of total RNA was performed using a NanoDrop 1000 Spectrophotometer (Thermo Scientific); following quantification, RNA samples were diluted to 10 ng/μl. qRT-PCR was performed using QuantiFast SYBR Green RT-PCR Kit (QIAGEN) according to the manufacturer’s recommendations in a 12.5 μl reaction volume. All reactions were performed using 7900HT Fast Real-Time PCR System machine (Applied Biosciences) with thermal cycling conditions as described [70]. As above, the relative fold change for each gene between CHIKV-infected and mock-infected was calculated using the Ct method after normalization to GAPDH [70]. See S1 Table for forward and reverse primers used.

### Viral RNA quantification

RNA was isolated using a PureLink RNA Mini Kit (Life Technologies) as described above. Absolute quantification of RRV RNA was performed as previously described [44]. Briefly, a sequence-tagged (small caps) RRV-specific RT primer (4415 5’-ggcagtatcgtgaattcgatgcAACACTCCCGTCGACAACAGA-3’) was used for reverse transcription. A tag sequence-specific reverse primer (5’-GGCAGTATCGTGAATTCGATGC-3’) was used with a RRV sequence-specific forward primer (4346 5’-CCGTGGCGGGTATTATCAAT-3’) and an internal TaqMan probe (4375 5’-ATTAAGAGTGTAGCCATCC-3’) during qPCR to enhance specificity. To create standard curves, 10-fold dilutions, from 10^8^ to 100 copies of RRV genomic RNAs, synthesized *in vitro*, were spiked into RNA from BHK-21 cells, and reverse transcription and qPCR were performed in an identical manner. The limit of detection was 100 genome copies.

### Statistical analysis

All data were analyzed using GraphPad Prism 5 software. Data were evaluated for statistically significant differences using a two-tailed, unpaired *t* test with or without Welch’s correction, a one-way analysis of variance (ANOVA) test followed by Tukey’s multiple comparison test, or a two-way ANOVA followed by a Bonferroni multiple comparison test. Comparison between the high viral load and low viral load group in the patient cohort was performed by two-tailed Mann Whitney *U* test. Similarly, for the *in vitro* infection studies, pair-wise comparison was performed using a two-tailed Mann Whitney *U* test. A *P*-value < 0.05 was considered statistically significant. All differences not specifically indicated to be significant were not significant (*P* > 0.05).

## Supporting Information

S1 Fig Association of Arg1 with disease progression. (A) Disease severity (defined in Materials and Methods) in HVL and LVL groups of patients during the acute phase of disease. Histogram shows the percentage of patients with mild (n = 12) or acute severe clinical phenotypes (n = 11). Statistical significance was measured using 2-sided Fisher exact test between the number of patients with severe disease in the different viral load groups. *** *P* < 0.0001. (B-E) Arg1 expression was compared between patients with severe (n = 11) or mild (n = 12) disease across the four time points analyzed: acute phase, early convalescent phase, late convalescent phase, and chronic phase. Comparison was performed by 2-tailed Mann Whitney *U* test. *** *P* = 0.002. Individual symbols represent individual patients at each time point. Horizontal bars represent the mean.
(TIF)

S2 Fig Supernatant from RRV-infected C2C12 muscle cells but not purified RRV induces Arg1 expression in J774 macrophages. (A) Immunoblot analysis of Arg1 expression in J774 macrophages following stimulation with culture supernatants from mock- or RRV-inoculated C2C12 muscle cells for 24 hours (n = 3/group). GAPDH was used as a loading control. (B) Arg1 and GAPDH band intensities were quantified, and Arg1 expression was normalized to GAPDH and expressed as the fold increase over GAPDH expression in mock-inoculated C2C12 muscle cell supernatant. * *P* < 0.05 as determined by ANOVA followed by Tukey’s multiple comparison test. (C-E) J774 macrophages were inoculated with C2C12 cell (“Sup”) or purified (“Pure”) RRV at a MOI of 10. Supernatant virus was ultra-centrifuged (“Spin”) to remove live virus or UV- or heat-inactivated prior to addition to macrophages. J774 macrophages were cultured in these conditions for 24 hours (n = 3/group) and then harvested for immunoblot analysis of Arg1. GAPDH was used as a loading control. Representative blot shown in (C); band intensities quantified in (E). Aliquots of each supernatant sample were used to quantify live virus present after each treatment via standard plaque assay; plaque assay results shown in (D). Dashed line in (D) indicates the limit of detection.
(TIF)

S3 Fig WT and LysMcre;Arg1^F/F^ mice have similar frequency and total number of CD4^+^ and CD8^+^ T cells in lymphoid tissues at 7, 10, and 14 dpi. Three-to-four week-old WT and LysMcre;Arg1^F/F^ mice were mock-inoculated (n = 11) or inoculated with 10^3^ PFU of RRV (n = 7–8 per time point, per genotype). At 7, 10, and 14 dpi, spleens and draining (left) popliteal LN (pLN) were harvested for FACS analysis. (A) Representative flow plots indicating the gating strategy to identify lymphocytes, CD3^+^CD8^+^ T cells versus CD3^+^CD8^-^ T cells, and CD3^+^CD4^+^ T cells in the spleen (left panel) and pLN (right panel) at 10 dpi. (B) Frequency of CD3^+^CD4^+^ T cells (left panel) and CD3^+^CD8^+^ T cells (right panel) in the spleens of WT and LysMcre;Arg1^F/F^ mice. (C) Total number of CD3^+^CD4^+^ T cells (left panel) and CD3^+^CD8^+^ T cells (right panel) in the spleens. (D) Frequency of CD3^+^CD4^+^ T cells (left panel) and CD3^+^CD8^+^ T cells (right panel) in the pLN of mice. (E) Total number of CD3^+^CD4^+^ T cells (left panel) and CD3^+^CD8^+^ T cells (right panel) in the pLN of mice. Data are represented as the arithmetic mean ± SEM and combined from two independent experiments. Each time point was individually evaluated for statistical difference by a two-tailed, unpaired *t*-test, and all were found to be not significant (*P* > 0.05).
(TIF)

S4 Fig IFN-γ expression in T cells sorted from the spleen of RRV-infected WT and LysMcre;Arg1^F/F^ mice. RT-qPCR analysis of IFN-γ expression in FACS-sorted T cells from the spleen of a mock-inoculated mouse (n = 1) or RRV-infected WT (n = 3) or LysMcre;Arg1^F/F^ mice (n = 3) at 10 dpi. Data are normalized to 18S rRNA levels, are expressed as the relative expression (*n*-fold increase) over expression in spleen T cells from the mock-inoculated mouse, and are represented as the arithmetic mean ± SEM.
(TIF)

S5 Fig Muscle-infiltrating CD4^+^ T cells from RRV-infected mice produce IL-10 and IFN-γ. Three-to-four week-WT mice (n = 4) were inoculated with 10^3^ PFU of RRV. On day 10 pi, quadriceps muscle tissue was dissected and digested, and the infiltrating leukocytes were re-stimulated *ex vivo* via incubation in the presence of anti-CD3 and anti-CD28 Abs. Cells incubated without anti-CD3 and anti-CD28 Abs (“No stim”) were used as a control for stimulation. Additionally, spleen cells from a mock-inoculated mouse were used as a control. (A) Representative flow plots demonstrating the gates delineating IL-10 and IFN-γ-producing cells after gating on CD4^+^ (top panel) or CD8^+^ (bottom panel) T cells. (B) Quantification of the percent of IL-10 and IFN-γ-double producing CD4^+^ and CD8^+^ T cells in muscle tissue with or without re-stimulation. Data are presented as mean ± SEM.
(TIF)

S6 Fig CD4^+^ T cells contribute to Arg1 induction in musculoskeletal tissues following RRV infection. WT mice were inoculated with 10^3^ PFU of RRV and treated with 200 μg of a CD4 T cell depleting Ab or a control Ab on day 4 pi (n = 6 mice/group). On day 7 pi, spleen and left quadriceps muscle tissues were harvested for flow cytometric analysis. Additionally, right quadriceps muscle tissue was harvested for analysis of RRV RNA levels and Arg1 mRNA levels. (A) Representative flow plots demonstrating the gating strategy to identify CD4^+^ T cells or CD11b^+^CD64^+^ macrophages in the spleen and muscle tissues of control Ab- and anti-CD4 Ab-treated mice. Before gating on CD4^+^ cells, cells were gated on CD3^+^B220^-^ cells in the top panel (spleen) and on CD3^+^CD64^-^ cells in the middle panel (muscle). (B-G) The percent (B, D) and number (C, E) of CD4^+^ T cells in the spleen (B, C) and quadriceps muscle (D, E) were quantified. The percent (F) and number (G) of CD11b^+^CD64^+^ macrophages in the muscle tissue were quantified. (H and I) The right quadriceps muscle was harvested for quantification of (H) Arg1 mRNA expression, which was normalized to 18S rRNA levels and expressed as the relative expression (*n*-fold increase) over expression in mock-inoculated mice, and (I) RRV genomes by absolute RT-qPCR. Data are combined from two independent experiments. (B-H) The data are presented as the arithmetic mean ± SEM. (I) Each symbol represents an individual mouse.
(TIF)

S7 Fig Ki67 staining of total and gp33-specific CD8^+^ T cells. Three-to-four week-old WT (n = 3) and LysMcre;Arg1^F/F^ (n = 3) mice were inoculated with 10^3^ PFU of RRV-LCMV. At 7 dpi, leukocytes from the spleen and quadriceps muscles (following enzymatic digestion) were isolated for flow cytometric analysis of Ki67 expression in bulk or gp33-specific CD8^+^CD3^+^ T cells.
(TIF)

S1 Table Primers used for gene expression analysis of human cells. (PDF)
