# Supplementary figures and images for: Myeloid Cell Arg1 Inhibits Control of Arthritogenic Alphavirus Infection by Suppressing Antiviral T Cells
Source: PLoS Pathog. 2015 Oct 5;11(10):e1005191. doi: 10.1371/journal.ppat.1005191 (PMC4593600; doi:10.1371/journal.ppat.1005191)

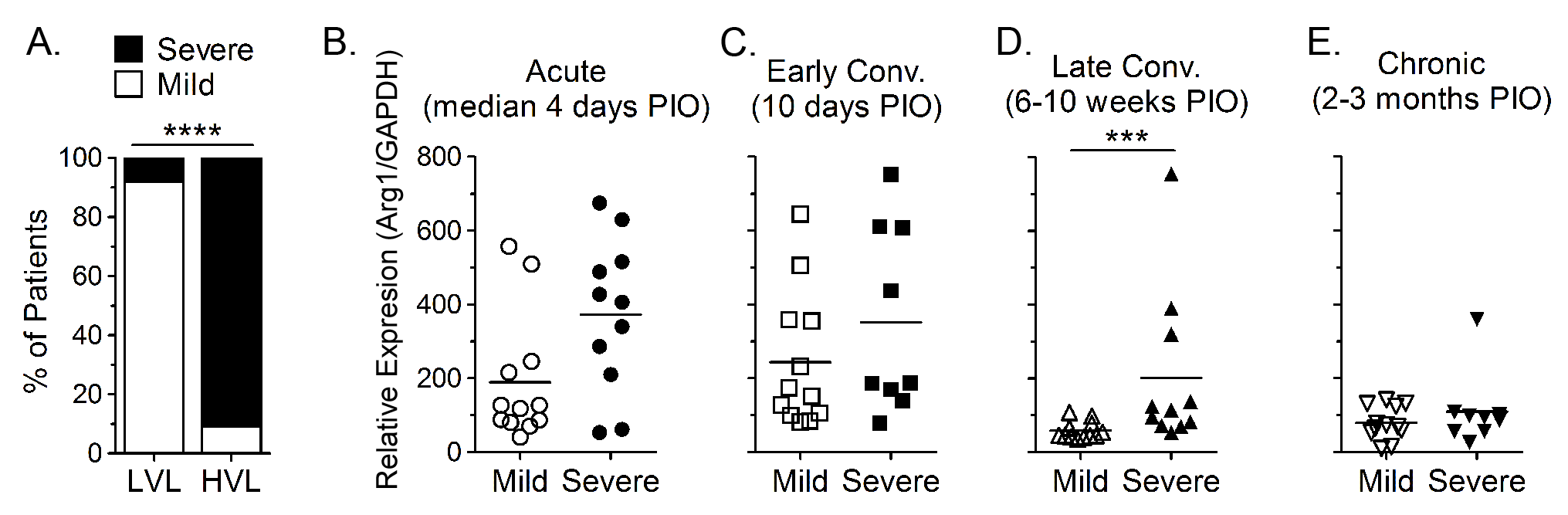

Supplement: S1 Fig — (A) Disease severity (defined in Materials and Methods) in HVL and LVL groups of patients during the acute phase of disease. Histogram shows the percentage of patients with mild (n = 12) or acute severe clinical phenotypes (n = 11). Statistical significance was measured using 2-sided Fisher exact test between the number of patients with severe disease in the different viral load groups. *** P < 0.0001. (B-E) Arg1 expression was compared between patients with severe (n = 11) or mild (n = 12) disease across the four time points analyzed: acute phase, early convalescent phase, late convalescent phase, and chronic phase. Comparison was performed by 2-tailed Mann Whitney U test. *** P = 0.002. Individual symbols represent individual patients at each time point. Horizontal bars represent the mean. (TIF) [file ppat.1005191.s001.tif]

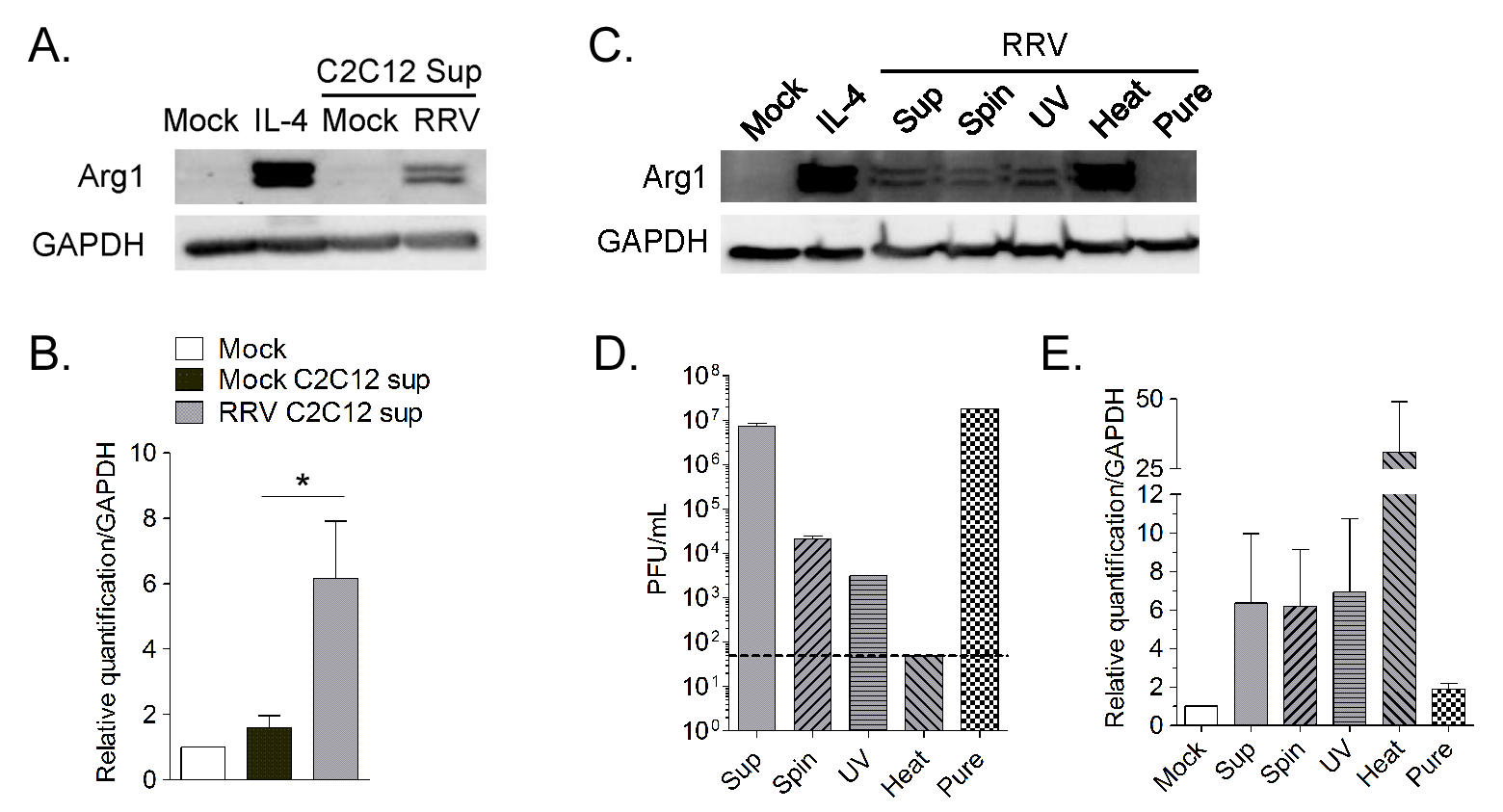

Supplement: S2 Fig — (A) Immunoblot analysis of Arg1 expression in J774 macrophages following stimulation with culture supernatants from mock- or RRV-inoculated C2C12 muscle cells for 24 hours (n = 3/group). GAPDH was used as a loading control. (B) Arg1 and GAPDH band intensities were quantified, and Arg1 expression was normalized to GAPDH and expressed as the fold increase over GAPDH expression in mock-inoculated C2C12 muscle cell supernatant. * P < 0.05 as determined by ANOVA followed by Tukey’s multiple comparison test. (C-E) J774 macrophages were inoculated with C2C12 cell (“Sup”) or purified (“Pure”) RRV at a MOI of 10. Supernatant virus was ultra-centrifuged (“Spin”) to remove live virus or UV- or heat-inactivated prior to addition to macrophages. J774 macrophages were cultured in these conditions for 24 hours (n = 3/group) and then harvested for immunoblot analysis of Arg1. GAPDH was used as a loading control. Representative blot shown in (C); band intensities quantified in (E). Aliquots of each supernatant sample were used to quantify live virus present after each treatment via standard plaque assay; plaque assay results shown in (D). Dashed line in (D) indicates the limit of detection. (TIF) [file ppat.1005191.s002.tif]

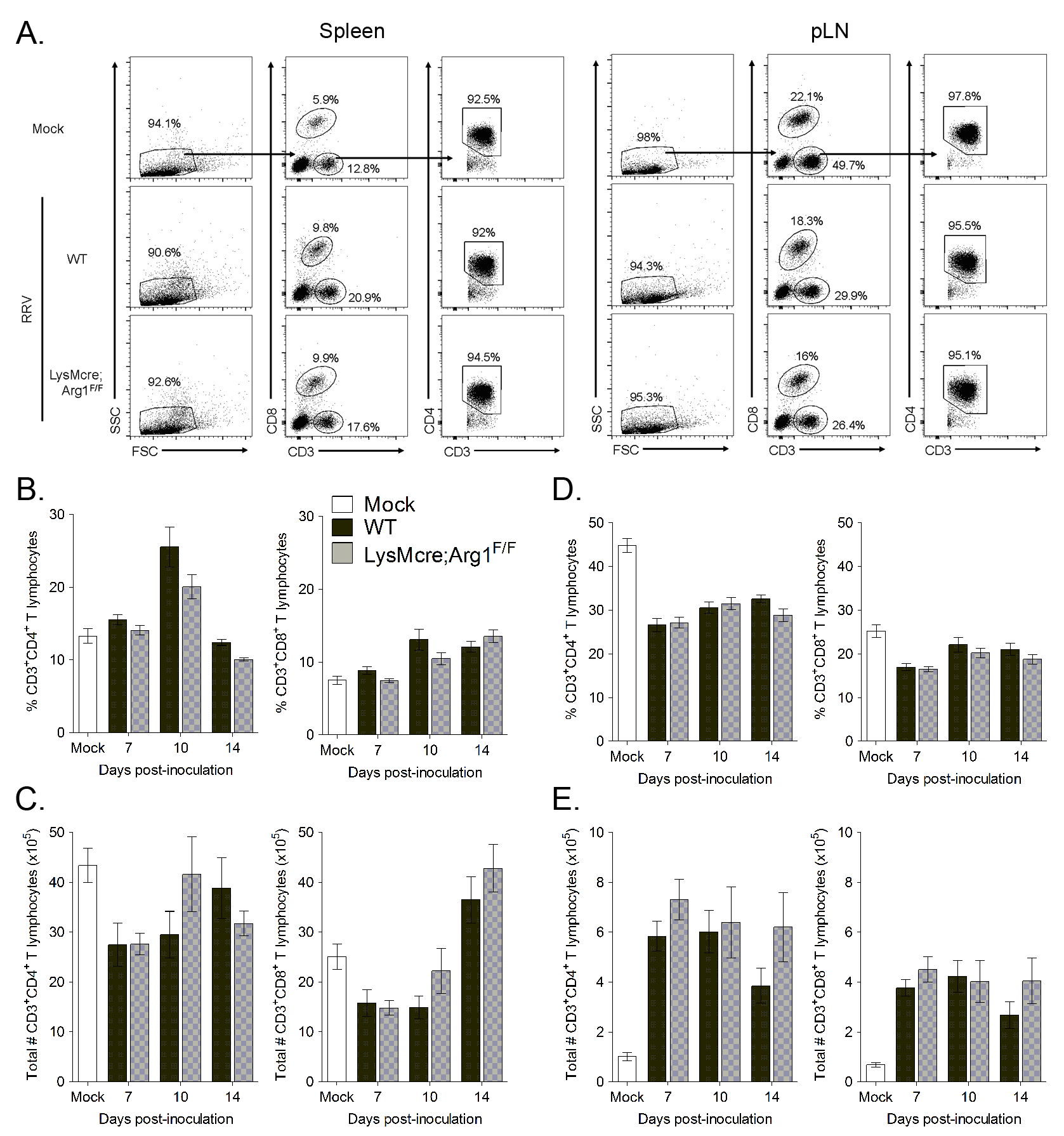

Supplement: S3 Fig — Three-to-four week-old WT and LysMcre;Arg1F/F mice were mock-inoculated (n = 11) or inoculated with 103 PFU of RRV (n = 7–8 per time point, per genotype). At 7, 10, and 14 dpi, spleens and draining (left) popliteal LN (pLN) were harvested for FACS analysis. (A) Representative flow plots indicating the gating strategy to identify lymphocytes, CD3+CD8+ T cells versus CD3+CD8- T cells, and CD3+CD4+ T cells in the spleen (left panel) and pLN (right panel) at 10 dpi. (B) Frequency of CD3+CD4+ T cells (left panel) and CD3+CD8+ T cells (right panel) in the spleens of WT and LysMcre;Arg1F/F mice. (C) Total number of CD3+CD4+ T cells (left panel) and CD3+CD8+ T cells (right panel) in the spleens. (D) Frequency of CD3+CD4+ T cells (left panel) and CD3+CD8+ T cells (right panel) in the pLN of mice. (E) Total number of CD3+CD4+ T cells (left panel) and CD3+CD8+ T cells (right panel) in the pLN of mice. Data are represented as the arithmetic mean ± SEM and combined from two independent experiments. Each time point was individually evaluated for statistical difference by a two-tailed, unpaired t-test, and all were found to be not significant (P > 0.05). (TIF) [file ppat.1005191.s003.tif]

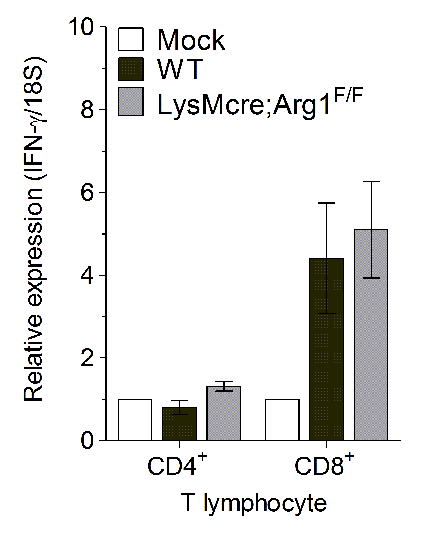

Supplement: S4 Fig — RT-qPCR analysis of IFN-γ expression in FACS-sorted T cells from the spleen of a mock-inoculated mouse (n = 1) or RRV-infected WT (n = 3) or LysMcre;Arg1F/F mice (n = 3) at 10 dpi. Data are normalized to 18S rRNA levels, are expressed as the relative expression (n-fold increase) over expression in spleen T cells from the mock-inoculated mouse, and are represented as the arithmetic mean ± SEM. (TIF) [file ppat.1005191.s004.tif]

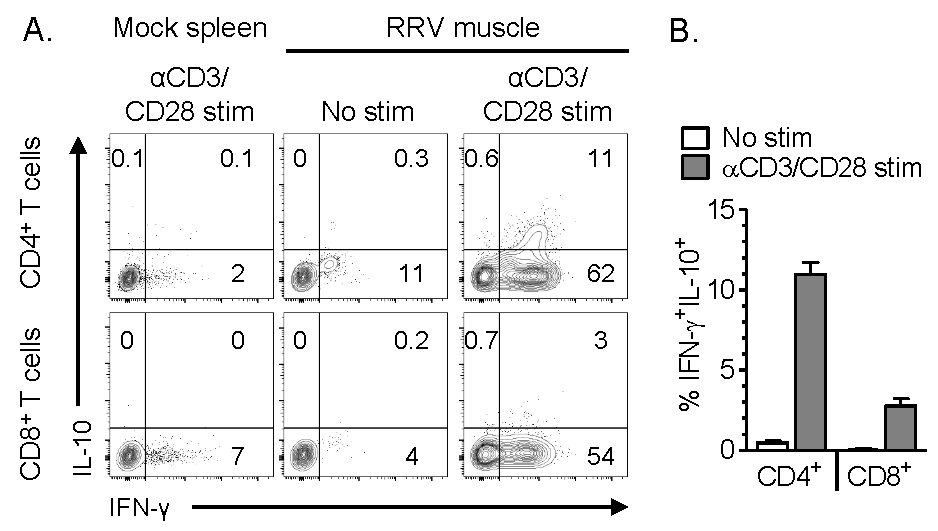

Supplement: S5 Fig — Three-to-four week-WT mice (n = 4) were inoculated with 103 PFU of RRV. On day 10 pi, quadriceps muscle tissue was dissected and digested, and the infiltrating leukocytes were re-stimulated ex vivo via incubation in the presence of anti-CD3 and anti-CD28 Abs. Cells incubated without anti-CD3 and anti-CD28 Abs (“No stim”) were used as a control for stimulation. Additionally, spleen cells from a mock-inoculated mouse were used as a control. (A) Representative flow plots demonstrating the gates delineating IL-10 and IFN-γ-producing cells after gating on CD4+ (top panel) or CD8+ (bottom panel) T cells. (B) Quantification of the percent of IL-10 and IFN-γ-double producing CD4+ and CD8+ T cells in muscle tissue with or without re-stimulation. Data are presented as mean ± SEM. (TIF) [file ppat.1005191.s005.tif]

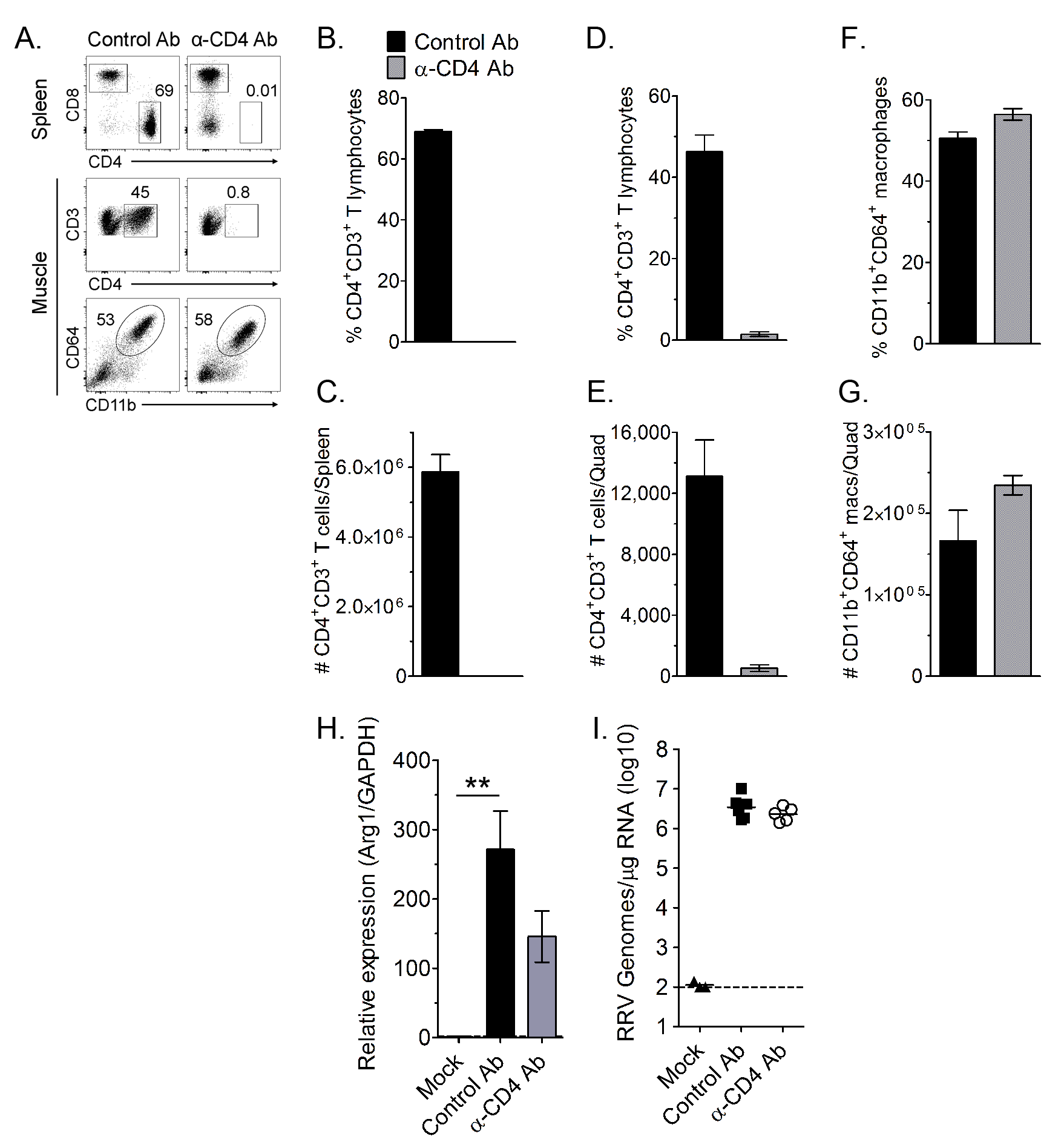

Supplement: S6 Fig — WT mice were inoculated with 103 PFU of RRV and treated with 200 μg of a CD4 T cell depleting Ab or a control Ab on day 4 pi (n = 6 mice/group). On day 7 pi, spleen and left quadriceps muscle tissues were harvested for flow cytometric analysis. Additionally, right quadriceps muscle tissue was harvested for analysis of RRV RNA levels and Arg1 mRNA levels. (A) Representative flow plots demonstrating the gating strategy to identify CD4+ T cells or CD11b+CD64+ macrophages in the spleen and muscle tissues of control Ab- and anti-CD4 Ab-treated mice. Before gating on CD4+ cells, cells were gated on CD3+B220- cells in the top panel (spleen) and on CD3+CD64- cells in the middle panel (muscle). (B-G) The percent (B, D) and number (C, E) of CD4+ T cells in the spleen (B, C) and quadriceps muscle (D, E) were quantified. The percent (F) and number (G) of CD11b+CD64+ macrophages in the muscle tissue were quantified. (H and I) The right quadriceps muscle was harvested for quantification of (H) Arg1 mRNA expression, which was normalized to 18S rRNA levels and expressed as the relative expression (n-fold increase) over expression in mock-inoculated mice, and (I) RRV genomes by absolute RT-qPCR. Data are combined from two independent experiments. (B-H) The data are presented as the arithmetic mean ± SEM. (I) Each symbol represents an individual mouse. (TIF) [file ppat.1005191.s006.tif]

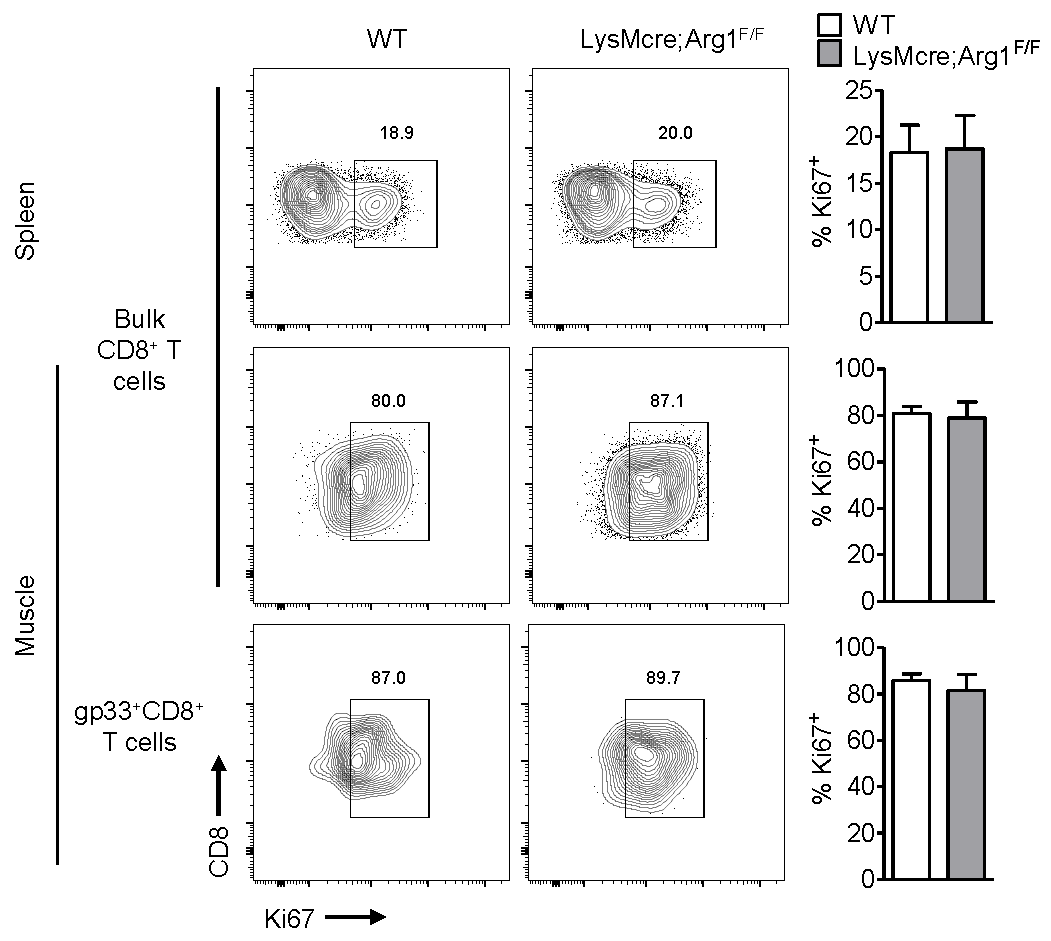

Supplement: S7 Fig — Three-to-four week-old WT (n = 3) and LysMcre;Arg1F/F (n = 3) mice were inoculated with 103 PFU of RRV-LCMV. At 7 dpi, leukocytes from the spleen and quadriceps muscles (following enzymatic digestion) were isolated for flow cytometric analysis of Ki67 expression in bulk or gp33-specific CD8+CD3+ T cells. (TIF) [file ppat.1005191.s007.tif]
