# Supplementary material for: Myeloid Cell Arg1 Inhibits Control of Arthritogenic Alphavirus Infection by Suppressing Antiviral T Cells
Source: PLoS Pathog. 2015 Oct 5;11(10):e1005191. doi: 10.1371/journal.ppat.1005191 (PMC4593600; doi:10.1371/journal.ppat.1005191)
Supplement: S1 Table — (PDF) [file ppat.1005191.s008.pdf]

Table S1. Primers used for gene expression analysis of human cells.

Gene	Forward Primer Sequence (5' → 3')	Reverse Primer Sequence (5' → 3')
GAPDH	CCACATCGCTCAGACACCAT	GGCAACAATATCCACTTTACCAGAGT
Arg1	TGGACAGACTAGGAATTGGCA	CCAGTCCGTCAACATCAAAACT
Nox1	CTGCTTCCTGTGTGTCGCAA	AGGCAGATCATATAGGCCACC
IL-6	CACAGACAGCCACTCACCTCTTCAGAACGA	ACCAGTGATGATTTTCACCAGGCAAGTCTC
IL-10	GACTTTAAGGGTTACCTGGGTTG	TCACATGCGCCTTGATGTCTG
TGF-β	GGCCAGATCCTGTCCAAGC	GTGGGTTTCCACCATTAGCAC
